# Genome-Wide Association Study of Golden Retrievers Identifies Germ-Line Risk Factors Predisposing to Mast Cell Tumours

**DOI:** 10.1371/journal.pgen.1005647

**Published:** 2015-11-20

**Authors:** Maja L. Arendt, Malin Melin, Noriko Tonomura, Michele Koltookian, Celine Courtay-Cahen, Netty Flindall, Joyce Bass, Kim Boerkamp, Katherine Megquir, Lisa Youell, Sue Murphy, Colleen McCarthy, Cheryl London, Gerard R. Rutteman, Mike Starkey, Kerstin Lindblad-Toh

**Affiliations:** 1 Science for Life Laboratory, Department of Medical Biochemistry and Microbiology, Uppsala University, Uppsala, Sweden; 2 Department of Veterinary Medicine, University of Cambridge, Cambridge, United Kingdom; 3 Broad Institute of MIT and Harvard, Cambridge, Massachusetts, United States of America; 4 Department of Clinical Sciences, Cummings School of Veterinary Medicine at Tufts University, North Grafton, Massachusetts, United States of America; 5 Animal Health Trust, Newmarket, United Kingdom; 6 Department of Clinical Sciences of Companion Animals, Utrecht University, Utrecht, The Netherlands; 7 Department of Veterinary Clinical Sciences Ohio State University, Columbus, Ohio, United States of America; 8 Veterinary Specialist Center De Wagenrenk, Wageningen, The Netherlands; University of Washington, UNITED STATES

## Abstract

Canine mast cell tumours (CMCT) are one of the most common skin tumours in dogs with a major impact on canine health. Certain breeds have a higher risk of developing mast cell tumours, suggesting that underlying predisposing germ-line genetic factors play a role in the development of this disease. The genetic risk factors are largely unknown, although somatic mutations in the oncogene *C-KIT* have been detected in a proportion of CMCT, making CMCT a comparative model for mastocytosis in humans where *C-KIT* mutations are frequent. We have performed a genome wide association study in golden retrievers from two continents and identified separate regions in the genome associated with risk of CMCT in the two populations. Sequence capture of associated regions and subsequent fine mapping in a larger cohort of dogs identified a SNP associated with development of CMCT in the *GNAI2* gene (p = 2.2x10^-16^), introducing an alternative splice form of this gene resulting in a truncated protein. In addition, disease associated haplotypes harbouring the hyaluronidase genes *HYAL1*, *HYAL2* and *HYAL3* on cfa20 and *HYAL4*, *SPAM1* and *HYALP1* on cfa14 were identified as separate risk factors in European and US golden retrievers, respectively, suggesting that turnover of hyaluronan plays an important role in the development of CMCT.

## Author Summary

Pet dogs develop many of the same diseases as humans. Hence, studying diseases in dogs can be valuable for learning about human conditions. The genetic structure caused by inbreeding within dog breeds has proven to be advantageous to map genetic diseases. Golden retrievers have a very high risk of developing mast cell tumours suggesting that there is a genetic background for this disease. In the present study we investigated genetic risk factors for this disease in golden retrievers. We identified three regions of the genome predisposing to the development of mast cell tumors. A candidate mutation in the *GNAI2* gene was found to change the form of this gene. The disease associated regions also harbour multiple hyaluronidase genes (*HYAL1*, *HYAL2* and *HYAL3* on cfa20 and *HYAL4*, *SPAM1* and *HYALP1* on cfa14) suggesting that turnover of hyaluronic acid plays an important role in the development of CMCT. Human mastocytosis shares many characteristics with canine mast cell tumours and we believe our findings can help clarifying the biology behind this disease in humans as well as identifying new therapeutic targets.

## Introduction

Mastocytosis is a term that covers a broad range of human conditions involving the uncontrolled proliferation and infiltration of mast cells in tissues. A common characteristic for these diseases is a high frequency of activating mutations in the *C-KIT* oncogene [1–3]. An intriguing feature of the disease spectrum is its ability to spontaneously resolve despite having a mutation in an oncogene, as seen commonly in the juvenile condition [4].

Mastocytosis in adults can be accompanied by additional haematological abnormalities and a reduced life expectancy [5]. In addition, the disease has major adverse effects on life quality for the affected individuals [6]. The most severe forms of mastocytosis, such as mast cell leukaemia, are considered very malignant and are associated with a poor prognosis due to a lack of treatment options [1,2].

CMCT shares many phenotypic and molecular characteristics with mastocytosis, including paraclinical and clinical manifestations, and a high prevalence of activating *C-KIT* mutations [7,8]. CMCT in dogs thus provides a good naturally occurring comparative disease model for studying mastocytosis [9,10]. As reported in humans [1,11,12], there is evidence for germ-line risk factors in dogs as specific breeds, including golden retrievers, Labrador retrievers, boxers and Chinese shar-pei, have a high frequency of CMCT [13,14]. Current treatment options for CMCT encompass radical surgery alone, or in combination with chemotherapy or radiotherapy. The tyrosine kinase inhibitors masitinib and toceranib are licensed for treatment of non-resectable CMCT [9]. Human mastocytosis on the other hand is often not responsive to tyrosine kinase inhibitors, as the common V816D *C-KIT* mutation makes this receptor resistant to the classical tyrosine kinase inhibitors [3].

The behaviour of mast cell tumours in dogs is difficult to predict and accurate prognostication is challenging despite current classification schemes based on histopathology [15,16]. Mastocytosis is commonly seen as a systemic or generalized cutaneous disease whilst CMCT are commonly solitary masses, which are localized in the skin. These spread via the lymphatic system to local lymph nodes and visceral organs such as liver spleen and kidneys [9]. Interestingly haematological spread of CMCT to the lungs has never been reported suggesting that these tumours spread solely via the lymphatic system rather than via a haematogenous route. In humans germline *C-KIT* mutations have been detected in familial mastocytosis [1]. There is no published research regarding predisposing germline mutations in dogs.

Modern dog breeds have been created by extensive selection for certain phenotypic characteristics. As a side effect, unwelcomed traits like diseases have also been enriched in different breeds. The recent bottlenecks during breed creation have given rise to extensive linkage disequilibrium (LD) within breeds [17]. Furthermore, as a result of the reduced genetic heterogeneity, the number of genetic risk factors is limited within a breed, thereby reducing the genetic complexity. These characteristics of the dog genome enable efficient disease mapping within a breed, using fewer markers and individuals compared to human studies and reducing the required sample numbers from thousands to hundreds [17,18].

The aim of this study was to identify genetic risk factors for CMCT in dogs. We carried out a genome wide association study (GWAS) comparing 107 healthy geriatric golden retrievers with 124 golden retrievers affected with CMCT. Samples were collected from Europe and the US, representing two populations from separate continents. This allowed us to identify two different significantly associated loci in the two populations each of which harbours three of the six hyaluronidase genes. Subsequent targeted sequencing and fine mapping was carried out in the associated regions and identified at least one compelling disease-associated variant.

## Results

### Genome-wide association testing

**Fig 1:**
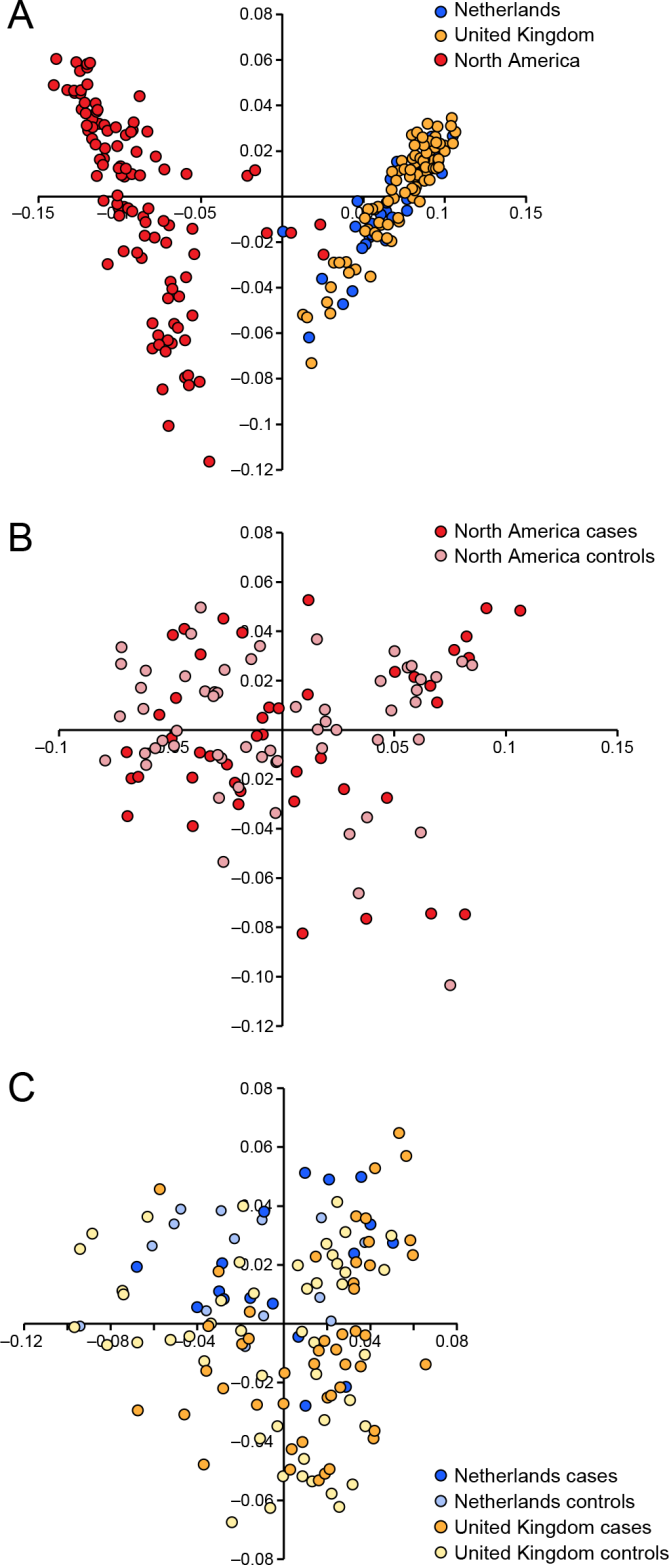
Multiple dimensional scaling plot visualising the genetic similarity between the individuals within the population using the two first principal components as calculated in PLINK. a) American and European data combined b) American data c) European data.

We conducted a case-control GWAS of 273 golden retrievers (GR) to find candidate regions associated with CMCT. After quality control and removal of related individuals, the final data set included a total of 124 cases and 107 controls with low levels of relatedness (genetic relationship matrix value <0.25) within the two subpopulations, and high genotype call rates (>90%). Two individuals were removed due to low genotyping rate, 40 individuals where removed due to high levels of relatedness. The multidimensional scaling plot (MDS) showed that the American and European GRs form two distinct clusters, indicating genetic differentiation between the populations on different continents (Fig 1A). This implies that the CMCT predisposition could have different genetic causes in the two populations. MDS plots for the two groups analysed separately indicated no outliers or substantial stratification within either cohort (Fig 1B and 1C).

**Fig 2:**
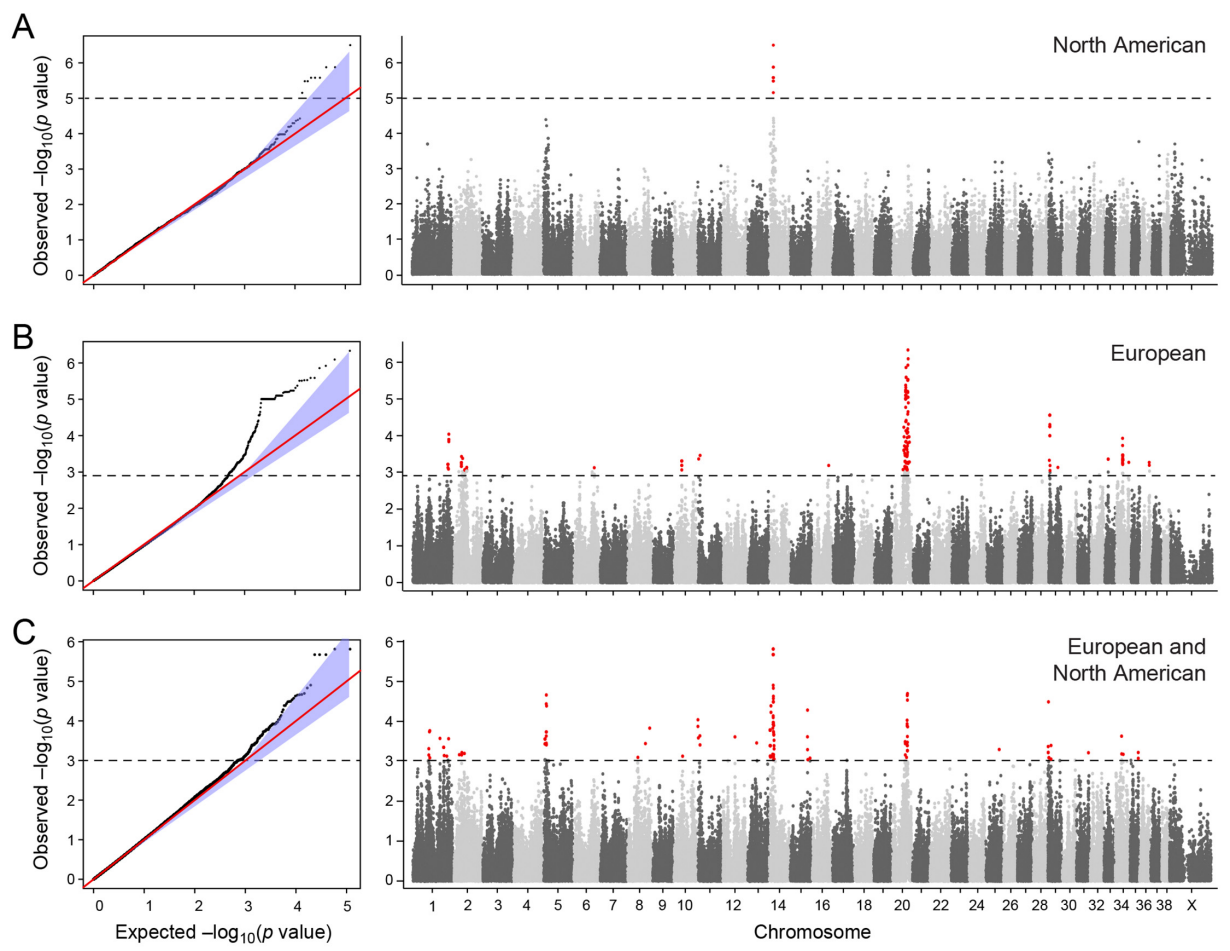
Manhattan plot showing the–log 10 p-values in relation to the chromosomes. a) American data b) European data c) American and European data combined. Q-Q plots showing the expected p-value in relation to the observed p-value for each GWAS analysis. Shaded area indicates 95% confidence interval. Stippled line marks nominal significance threshold.

The two cohorts were first analysed separately, and then together using a mixed model approach. Essentially no genomic inflation was detected in the US and EU analysis, as evidenced by the QQ plots and genomic inflation factor (λ = 1.01 for both EU and US respectively, Fig 2). The Manhattan plots for the two different populations (Fig 2A and 2B) showed one major associated locus for each population. However, the two loci were not overlapping, but are on two different chromosomes (cfa14 and 20), suggesting that different genetic risk factors are influencing the two populations of GRs.

**Fig 3:**
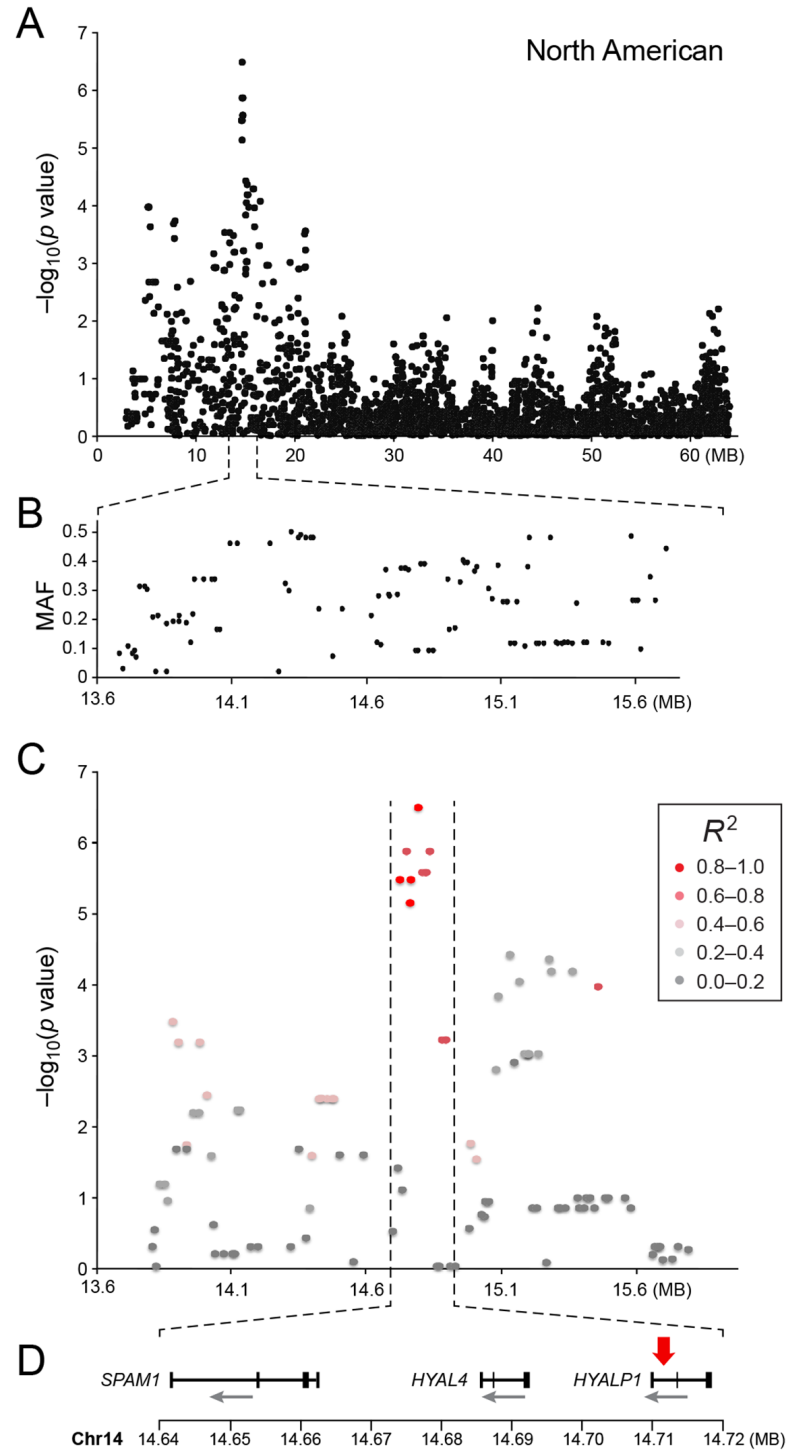
a) Close up view of the most associated region from the American GWAS analysis. b) Minor allele frequency of the associated region. c) Further close up of the associated peak showing the LD structure of the SNPs in the area relative to the most associated SNP. d) Close up view of the genes located in the area of the associated haplotype. Red arrow indicates the location of the most associated SNP.

The American GR association analysis (n_cases_ = 59, n_controls_ = 45) resulted in one significantly associated region on cfa14 (nominal significance threshold at -log p>5.0, based on the deviation in the QQ plot, Fig 2A). Nine SNPs were found to be significantly associated with CMCT (Fig 3), with the strongest association (p = 3.2x10^-7^, p_perm_ = 0.03) at CanFam2.0 cfa14:14,714,009 bp conferring a substantial risk effect (OR = 5.3). The risk allele frequency for the most associated SNP was 0.86 in cases and 0.53 in control GRs, and all cases except for one carry at least one copy of the risk genotype (S1A Fig). However, this case is heterozygous for the European GR risk alleles. The five SNPs with the strongest association are presented in Table 1, and all significantly associated SNPs are listed in S1 Table. All of the significant SNPs on chromosome 14 show high LD with the most associated SNP (Fig 3C) and nine SNPs form a risk haplotype spanning 111 kb (14.64–14.76 Mb) containing only three genes; *SPAM1*, *HYAL4* and *HYALP1*. Notably, the genes are all hyaluronidase enzymes. The top SNP is located within the 2nd intron of the *HYALP1* pseudogene.

**Table 1:** Top-ranking GWAS SNPs from European and United States data. Table showing the 5 most associated GWAS SNPs from the European and American analysis.

	SNP	Chr	Position	Allele	*P* U.S.	*P* Eur.	*P* Comb.	*P*perm 10,000	OR	AFa	AFu
European (Netherlands and United Kingdom)	BICF2S22934685	20	42,547,825	C/T	0.72	1.4×10^−6^	0.0042	0.034	6.2	0.91	0.64
	BICF2P1444805	20	42,957,449	A/G	0.90	2.6×10^−6^	0.0067	0.078	6.5	0.94	0.70
	BICF2P299292	20	48,377,580	A/C	0.45	1.2×10^−6^	2.2×10^−5^	0.085	4.0	0.65	0.31
	BICF2P301921	20	48,599,799	A/C	0.58	4.3×10^−7^	2.2×10^−5^	0.022	4.1	0.65	0.31
	BICF2P623297	20	49,201,505	G/A	0.54	8.1×10^−7^	2.0×10^−5^	0.037	4.2	0.63	0.29
United States	BICF2P867665	14	14,714,009	G/T	3.2×10^−7^	0.27	1.2×10^−5^	0.030	5.2	0.86	0.53
	BICF2G630521572	14	14,670,361	T/C	1.3×10^−6^	0.09	1.5×10^−6^	0.099	4.2	0.77	0.44
	BICF2G630521696	14	14,756,089	G/A	1.3×10^−6^	0.09	1.5×10^−6^	0.099	4.2	0.77	0.44
	TIGRP2P186605	14	14,727,905	G/A	2.6×10^−6^	0.09	2.1×10^−6^	0.17	4.0	0.76	0.44
	BICF2G630521678	14	14,740,313	A/G	2.6×10^−6^	0.09	2.1×10^−6^	0.17	4.0	0.76	0.44

P US = p-values for the American analysis, P EU = p-values for the European analysis, P-comb = p-value for the combined European and American analysis, Pperm 10,000 = permutated p-value 10,000 permutations, OR = odds ratio, AFa = allele frequency of risk allele in affected individuals in respective populations, AFu = allele frequency of risk allele in control individuals in respective populations.

**Fig 4:**
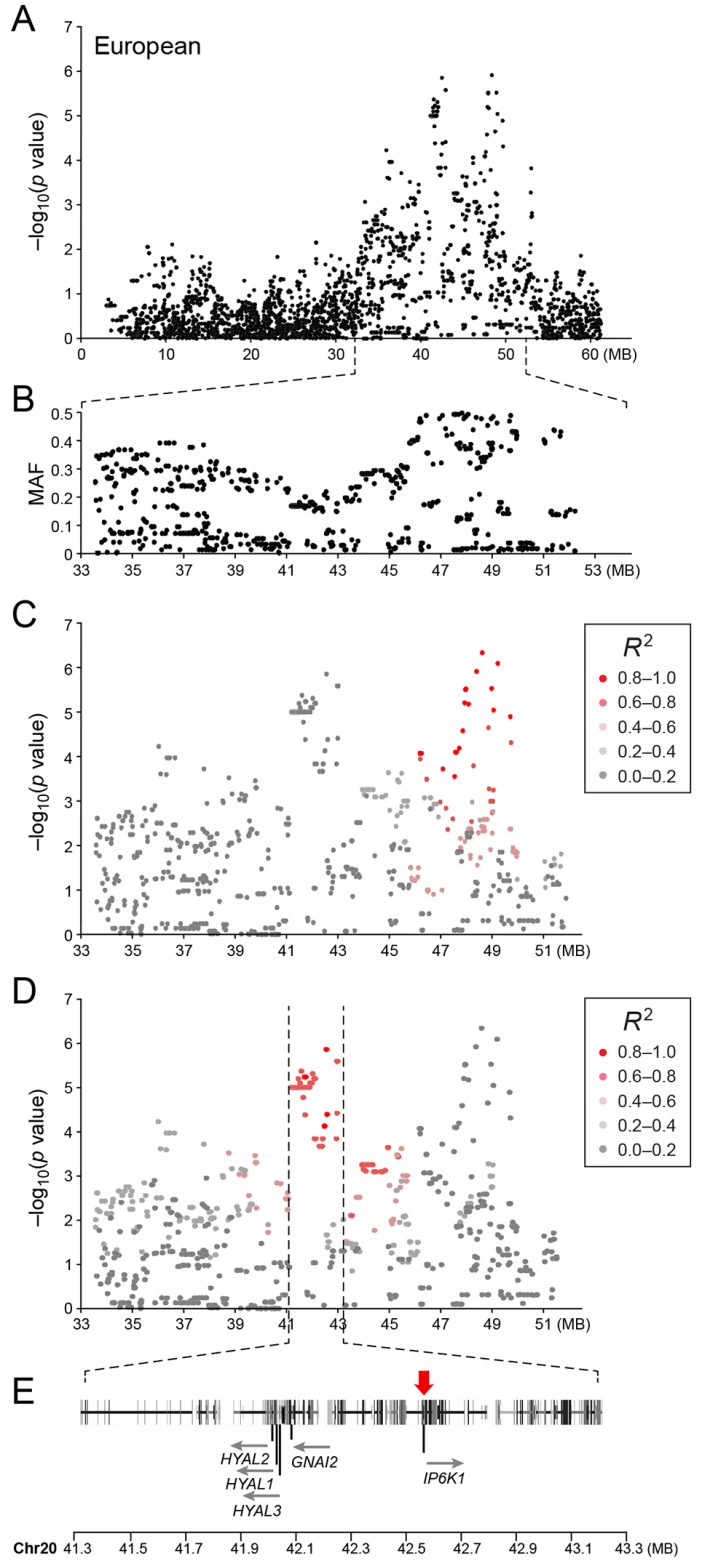
a) Close up view of the most associated region from the European GWAS analysis. b) Minor allele frequency of the associated region. c) Further close up of the associated region showing the LD structure of the SNPs in the 48 MB locus relative to the most associated SNP in the locus. d) Further close up of the associated peak showing the LD structure of the SNPs in the 42 MB locus relative to the most associated SNP in the locus. e) Close up view of the genes located on the associated haplotype in the 42MB region. Red arrow indicates the location of the most associated SNP in the region.

In the European population (n_cases_ = 65, n_controls_ = 62), chromosome 20 showed the strongest association, while ten chromosomes showed nominal significance (-log p>2.9, based on the QQ-plot, Fig 2B). The nominal significance determines that there are associated SNPs below the nominal significance threshold, however not all p-values below this level are significant. The strong signal from chromosome 20 suggest that this region has a high probability of being associated, while only some of the less significant regions may be truly associated. On chromosome 20, 167 SNPs spanning 20 Mb (33.9Mb–53.1Mb) showed nominal significance. They form two major loci at 42Mb (most associated SNP p = 1.4x10^-6^, p_perm_ = 0.039, OR = 6.3, cfa20:42,547,825 bp) and 48Mb (strongest associated SNP p = 4.3x10^-7^, p_perm_ = 0.022, OR = 4.1, cfa20:48,599,799 bp). Analysis of the LD in the area shows that the top SNPs in each region are in high LD with nearby SNPs, but low LD (r^2^<0.2) with SNPs in the other peak (Fig 4). The risk allele frequency for the SNP at 42Mb is high, with an allele frequency of 0.92 in cases and 0.64 in controls. However, the risk allele at 48Mb is less common, with a frequency of 0.65 in cases and 0.31 in controls. The discrepancy in allele frequencies supports the inference that the associated loci are independent and could harbour separate risk factors for CMCT. The differences in risk haplotype frequencies are also evident from the minor allele frequency plot (Fig 4B). The minor allele frequency is reduced around 42Mb, indicating a reduction in genetic diversity, possibly due to selection in that region. The candidate region contains nearly 500 genes and corresponds to human chromosome 3p21, a region often affected by chromosomal abnormalities in cancer [19]. The most associated SNP at 48Mb falls between the *MYO9B* and *HAUS8* genes and, interestingly, there is a cluster of hyaluronidase genes (*HYAL1*, *HYAL2* and *HYAL3*) positioned within the association locus at 42Mb. As expected the GWAS analysis of the full cohort (n_cases_ = 124, n_controls_ = 107) showed partial overlap with the results from the American and European subsets and resulted in a decrease in the p-values for both cfa 20 and 14 (Fig 2C). Full cohort analysis resulted in a residual genomic inflation after correction (λ = 1.03).

### Sequence capture and fine mapping

Hybrid capture and subsequent Illumina sequencing of the most associated GWAS regions were performed in order to identify all variants in the regions. In total 3,357 variants were identified in 0.9 Mb on chromosome 14 and 16,972 variants were identified in 5.5 Mb on chromosome 20, including both INDELs and SNPs. The 132 SNPs selected for fine mapping were located on cfa 14 in the 14 Mb region (30 SNPs), on cfa 20 in the 42Mb region (38 SNPs), and on cfa 20 in the 48 Mb region (64 SNPs). Fine mapping was performed on DNA from 384 dogs. Twenty-eight SNPs were filtered out due to low genotyping rate (>0.7). This high number was due in part to the presence of repeat elements or duplicated sequences in the proximity of the SNP, allowing primers to align to more than one region in the genome. Six SNP’s were not polymorphic in the sequencing data but were chosen for genotyping because of their interesting location. These SNPs were excluded from further association analysis as all genotyped individuals carried the alternative allele. Five individuals were removed for a low genotyping rate (<50%) and 4 control individuals were removed, as they were no longer deemed suitable as controls due to development of a secondary malignancy, these individuals were not included in the original GWAS analysis. After filtering, DNA samples from 375 individuals remained for analysis, comprising 245 American dogs (100 cases, 145 controls) and 130 European dogs (65 cases, 65 controls). The DNA samples were from dogs included in the GWAS analysis and additional American individuals. Related individuals were not excluded from the analysis.

The American GR population showed the strongest fine mapping association to a SNP at cfa14: 14,644,897 (p_iPLEX(US)_ = 6.4x10^-8^, p_perm_ = 9.0 x10^-6^). This is one of the original GWAS SNPs (p_GWAS(US)_ = 3.3x x10^-6^) that formed part of the original GWAS risk haplotype. Six SNPs showed a strong association (p <10^−7^) and formed a high LD haplotype narrowing the associated risk haplotype to a 60kb region encompassing the *HYAL4* and *SPAM11* genes (S2 and S4A Figs). Among the most associated SNPs in the *HYAL4* gene were three coding SNPs (cfa14:14,685,543, cfa 14:14,685,602, cfa14:14,685,771), of which 14:14,685,543 was a GWAS SNP. All three mutations in the *HYAL4* gene cause amino acid changes, which are predicted as benign (score < 0.2, PolyPhen-2). A less associated coding SNP in the *SPAM1* gene (cfa14:14,653,880) was also found, which causes an amino acid change, which is predicted to be damaging (score 0.91, PolyPhen-2). This mutation is more prevalent in cases than controls, although the SNP is not in high LD with the risk haplotype. The most associated GWAS SNP from the American analysis (p_GWAS(US)_ = 3.2x10^-7^, cfa14:14,714,009 bp), was included in the fine mapping (p_IPLEX(US)_ = 4.08x10^-6^), and was found to be the 7th most associated SNP (S2 Table and S4A Fig).

An outstanding causal variant for the cfa14 14Mb association with CMCT in US GRs has yet to be identified. However, the associated haplotype traversing the region could be used as a predictive marker for development of CMCT in US GR dogs. Only a subset of the variants identified from the hybrid capture, were included in the fine mapping. Some coding SNPs in the *SPAM1* and *HYAL4* genes were not included in the fine mapping due to constraints of the design.

The European population showed the strongest fine mapping association to a SNP (cfa20:42,080,147, p = 2.0x10^-15^, p_perm_ <0.00001). This SNP showed an association p = 7.0 x10^-4^ when the US dogs were analysed alone. When the US and European data were analysed together a lower p-value was seen (cfa20:42,080,147, p = 2.2x10^-16^, p_perm_<0.00001). Interestingly this SNP is not in LD with the surrounding SNPs and appears to be a recent mutation, which is present only on the risk haplotype (S4B Fig). All but two European cases carry a copy of the risk allele (allele frequencies: cases = 0.83, controls = 0.35). However, the allele is rare in both cases and controls in the US population (allele frequencies: cases = 0.07, controls = 0.01) (S2 and S3 Tables and S5A Fig). The SNP is a synonymous SNP located at the final position in exon 3 of the Guanine Nucleotide Binding Protein (G Protein) Alpha Inhibiting Activity Polypeptide 2 (*GNAI2*). This changes the last base from a guanine (G) to an adenine (A). A splice site prediction software (Alternative Splice Site Predictor [20]) predicted this variant to change the site from a constitutive donor splice site to a suboptimal donor site.

The second most associated SNP (cfa20:42,131,456 p = 7.7x10^-6^) in the European analysis forms a long haplotype with 12 other fine mapping SNPs in high LD, traversing the region across the hyaluronidase genes in the 42MB region (S4C Fig). This SNP is a conserved, coding synonymous SNP located in exon four of the *GNAT1* gene (amino acid D98).

The GWAS identified the strongest association to the cfa20 48Mb region in the European population. In the fine mapping the association to the cfa20 48Mb region is less noteworthy than the association to the cfa20 42Mb region. The two most associated SNPs (cfa20:48,599,799 and cfa20:49,201,505) from the GWAS were included in the fine mapping. The SNP cfa20:49,201,505 was found to have the lowest p value of the SNPs located in the 48Mb region p_iplex_EU =_ 2.1x10^-5^. This SNP was found to be the 4th most associated SNP in the European analysis. S3 Table summarises the results of association testing in the European population and the combined European and US population.

### Phenotypic correlation with risk genotype

Phenotypic data such as age of onset, mast cell tumour grade and disease outcome was available for some of the cases. As the samples were collected from multiple institutions and the format of reporting was variable. For the European population the mean age of disease onset varied significantly between dogs which were homozygous versus heterozygous for the *GNAI2* risk SNP. Mean age of onset homozygous: 5.6 ± 0.4, n = 43, heterozygous: 7.6 ± 0.5, n = 17, p = 0.0073 as determined by unpaired t-test statistics. Only two dogs were homozygous for the protective allele and hence this was too little for statistical analysis. For the United States population age of onset was only available for 15 dogs and hence reliable test statistics could not be performed.

### Hyaluronan staining in normal and mast cell tissue

The GWAS analysis suggested that the breakdown of hyaluronic acid may play a role in the development of CMCT. We hence wanted to evaluate if hyaluronan formed part of the extracellular matrix of CMCT. Immunohistochemistry was performed on 12 mast cell tumour samples from GRs and on normal control tissues (skin and pannicular fat) from an unaffected dog. As seen in S9A and S9B Fig, immunohistochemistry confirmed that the mast cell cytoplasmic membrane does stain intensely positive for hyaluronan confirming that indeed hyaluronan forms part of the mast cell cytoplasmic membrane. Dermal and pannicular collagen directly adjacent to the mast cell tumour is increased and showed more intense staining of the intercellular/extracellular matrix. In comparison, normal dermal and pannicular tissue stained only mildly positive for hyaluronan, except for the basal membranes of the epidermis, which is known to contain hyaluronan, and which stained intensively positive (S9C and S9D Fig).

### Identification and confirmation of an alternative splice site in the *GNAI2* gene

**Fig 5:**
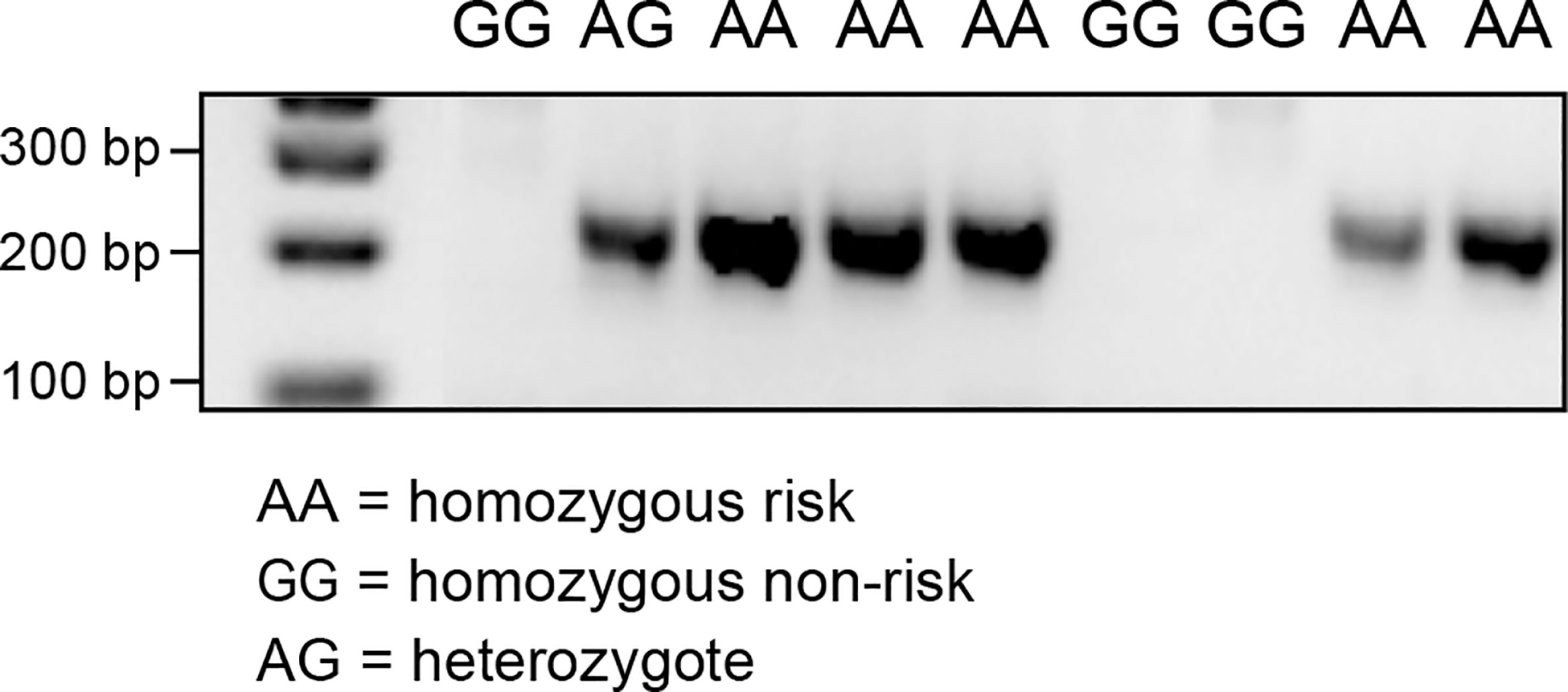
PCR products confirming the alternative splice site in the individuals carrying the A allele on CFA20: 42080147. PCR was performed using primers specific for the alternative splicing, excluding exon 3.

RNA sequencing of a CMCT and a normal cutaneous tissue sample was carried out in order to identify alternative transcript isoforms, and to evaluate which genes are expressed in CMCT. The CMCT was borne by a GR known to be homozygous for the variant SNP at cfa20:42,080,147 in the *GNAI2* gene that is predicted to change the site from a constitutive donor splice site to a suboptimal donor site. An alternative isoform of the *GNAI2* gene was identified by visual examination of the TopHat [21] output in IGV. This alternative isoform skips exon 3, showing that the identified cfa20:42,080,147 variant does change the splicing at this site. Quantitative PCR was performed on cDNA samples from 9 GRs using splice-specific primers traversing both the normal and the alternative splice site (S6 and S7 Figs). As seen in Fig 5, PCR products for the alternative splice form, were only detected in the individuals carrying one or more copies of the A allele at the cfa20:42,080,147 position. On average, the wild-type isoform was expressed at a 6.9-fold greater level than the alternatively spliced version as calculated by the difference in CT values between the normal and alternative splice products in homozygous individuals. The alternative splicing, splices out of frame and is predicted to produce a truncated protein, changing the open reading frame from 356 aa to 109 aa.

The RNA sequencing data also confirmed that GRs express the hyaluronidase genes. The *HYAL1*, *HYAL2*, *HYAL3* and *SPAM1* genes were expressed in both the CMCT and marginal normal tissue, but the *HYAL4* and *HYALP1* genes showed no evidence of expression in either tissue.

## Discussion

We identified genetic associations between CMCT and three different loci for American and European GR populations. The American population had the strongest association to a locus on chromosome 14 in which the hyaluronidase genes *HYAL4* and *SPAM1*, and the pseudogene *HYALP1*, are located. The European population showed association to two separate regions on chromosome 20 located around 42Mb and 48Mb, of which the 42Mb region harbours the remaining hyaluronidase genes *HYAL1*, *HYAL2* and *HYAL3*.

Sequence capture of the associated regions, in a small subset of individuals, identified thousands of variants, of which a large subset in each region followed the GWAS predicted risk haplotype. Fine mapping with additional markers narrowed down the risk haplotype on chromosome 14 from 111kb to a 60kb region, harbouring the *SPAM1* and *HYAL4* genes. The strongest associated SNP from the fine mapping on cfa14, was one of the original GWAS SNPs, BIC2G630521696 cfa14:14,756,089. Although the majority of candidate variants were included in the fine mapping in this region, including three coding SNPs in the *HYAL4* gene and one coding SNP in the *SPAM1* gene, there were several candidate variants, which could not be included in the fine mapping. For instance, a non-synonymous coding SNP in the *SPAM1* gene and a SNP 227bp downstream of the *SPAM1* gene could not be evaluated. Based on this and the strong LD in the region we have identified a predisposing haplotype, but not the causative variant yet. Future work will focus on further restriction of the identified risk haplotype with the aim to pinpoint the causative variant that could potentially be used to predict risk for CMCT development.

In the European population, the two associated loci located at 42 and 48Mb on cfa 20, respectively, were shown to be independent. Low LD was found between the SNPs in the two regions and the allele frequencies were also different, which suggest that they are independent and potentially contain separate risk factors for CMCT. Fine mapping of the relatively large 48Mb region did not narrow down the risk haplotype and the most associated SNP in the region was BICF2P623297 cfa20:49,201,505, which was one of the original GWAS SNPs. Fine mapping of the 42Mb region identified the cfa20:42,080,147 SNP strong association with CMCT in the European population and also mild association in the US GRs where it was rare. This SNP was not in LD with any of the other SNPs in the region although it was located only on the GWAS risk haplotype and hence it is likely to be a recent mutation on the risk haplotype. This SNP causes a change in a splice site in exon 3 of the *GNAI2* gene resulting in the production of an alternative transcript isoform through the skipping of the third exon. This alternative splice isoform splices out-of-frame and therefore introduces a stop codon at amino acid position 109 resulting in a truncated *GNAI2* protein. Expression analysis of the *GNAI2* gene, using splice specific primers, confirmed the presence of alternative splice isoforms in individuals carrying the mutation. As the normal isoform for this gene is still expressed in individuals carrying the risk genotype it is not known what effect the alternatively spliced protein will have. *GNAI2* belongs to a group of proteins, which regulate receptor signalling by controlling adenylyl cyclase activity [22]. *GNAI2* has frequently been linked to cancer and is also known as the gip2-oncogene [23]. Suppression of *GNAI2* has been detected in ovarian cancer [24] and somatic *GNAI2* mutations have been identified in diffuse large B-cell lymphoma [25]. *GNAI2* is highly expressed in the human mastocytoma cell line HMC-1 (The Human Protein Atlas [26]), as confirmed by both antibody staining and mRNA expression. We also found that *GNAI2* was expressed in a GR mast cell tumour, and marginal normal tissue. We have not been able to determine in this study whether the truncated *GNAI2* protein has a direct detrimental effect, or whether a loss of function from the truncation results in reduced regulation of adenylyl cyclase and increase activity of certain cellular pathways. This question warrants further study.

The coincidence that the two loci identified in the American and European GR populations, each contain three of the known six hyaluronidase genes, has lead us to hypothesize that hyaluronan turnover could play a role in CMCT predisposition. Interestingly, the Chinese shar-pei dog, which has an increase in hyaluronan accumulation in the skin due to a duplication upstream of the *HAS2* gene [27], also has an increased risk of developing CMCT. Furthermore, the naked mole rat has a decreased activity of hyaluronan degrading enzymes, which is believed to contribute to its longevity and resistance to cancer [28].

It is not known whether the *GNAI2* variant, located almost 20kb away from the hyaluronidase cluster, also has an effect on the hyaluronidase genes, or if this is a separate risk factor recently acquired by the risk haplotype. It has been shown that the human region (3p21.31), which is autologous to the canine cfa20 42Mb region, has been under selection in East Asians. This is thought to be due to *HYAL2* and its functions in the cellular response to UV-B light exposure [29]. It is possible that the low minor allele frequency in the hyaluronidase gene-containing areas of the genome in the golden retriever is a sign of selection. We speculate that the selection could be related to reproductive fitness, as the hyaluronidase genes play a role in reproduction [30,31].

Early studies of mast cells suggested that these cells contain hyaluronan. A correlation between the presence of hyaluronan and mast cells has been documented, and hence it was natural to believe that mast cells were the source of the hyaluronan [32,33]. However, later studies show that there is no evidence of mast cells producing hyaluronan *in vitro* [34]. Hyaluronan is broken down on the cell surface to smaller molecules by hyaluronidase [35], and the fragmented hyaluronan is then taken into the lysosomes of the cell and there further broken down by intracellular hyaluronidase. We find it plausible that mast cells interact with hyaluronan and play a role in hyaluronan turnover. Concordant with that, our CMCT RNA sequencing demonstrated expression of all the hyaluronidase genes except *HYAL4* and *HYALP1*. The breakdown products of hyaluronan, known as low molecular hyaluronan, have both pro-inflammatory and pro-oncogenic effects [35]. Studies in rats showed that intravesical injection of hyaluronidase resulted in inflammation and an increase in the number of activated mast cells, suggesting a direct role between hyaluronan break down products and mast cell activation and migration [36,37]. *In vitro* studies have also shown that mast cell proliferation can be inhibited by hyaluronan excreted by co-cultured cells [34]. Furthermore, mast cell secretion products have been shown to regulate hyaluronan secretion from other cells [38]. Mast cells also express the CD44 hyaluronan receptor on their cell surface [39]. Our immunohistochemical staining showed that hyaluronan forms part of the extracellular matrix in mast cell tumours and so likely interacts with the CD44 receptor. The interaction between CD44 and hyaluronan is known to promote both transformation and metastasis of cancer cells. Together these factors suggest that alterations in hyaluronan turnover could play a role in CMCT development.

Based on our data it appears possible that alterations in both the *GNAI2* and hyaluronidase genes play a role in mast cell tumour development. The association to regions containing hyaluronidase genes on both chromosome 14 and 20 together with the much stronger association to a novel variant in the *GNAI2* gene supports both findings. Still more work is required to validate and explore the functional consequences of these candidate genes. Many candidate variants were identified from the sequence capture and only a small subset were included in the fine mapping, which is a major limitation in this study. Many variants need to be studied in more detail to determine their effects.

The dog has proven to be a good model for many human disorders. Similarities between CMCT and human mastocytosis suggest that genes and genetic pathways altered in CMCT could also play a role in human mastocytosis. We will continue to evaluate the role of the *GNAI2* and the hyaluronidase genes in CMCT and hope that these investigations will help shed a light not only on CMCT, but also on human mastocytosis leading the way to a better understanding of the disease and potential new drug targets.

## Materials and Methods

### Samples

A total of 127 golden retriever (GR) samples were collected in the United States (70 cases and 57 controls), 113 in the United Kingdom (53 cases and 60 controls) and 33 in the Netherlands (18 cases and 15 controls). All samples were collected between year 2000 and 2013. The samples collected in the United States were collected from all over the United States. These samples were all collected by a veterinary professional and were submitted to the BROAD institute either by the veterinarian or by the dog owner. Samples collected in the UK were primarily collected at the Animal Health Trust (AHT). A subset of UK samples were collected by veterinarians or dog owners not affiliated with the AHT. Samples collected in the Netherlands were collected at either Utrecht University clinic of Companion Animals or Veterinary Specialist Center De Wagenrenk. Cases were diagnosed with CMCT by histopathology or cytology. Data was collected when available regarding age of onset, and grading of the mast cell tumour. Control dogs were unaffected by any form of cancer, and were over 7 years old. For the American controls, phoning the owners bi-yearly provided follow up health information. Genomic DNA was extracted from whole blood (240 samples) or buccal swabs (33 samples) using the QIAamp DNA Blood Midi Kit (QIAGEN), the Nucleon® Genomic DNA Extraction Kit (Tepnel Life Sciences), by phenol chloroform extraction, or by salt extraction [40].

### Genome-wide association mapping

Illumina 170K canine HD SNP arrays were used for the genotyping of approximately 174,000 SNPs with a mean genomic interval of 13kb. Genotyping of the European samples was performed at the Centre National de Genotypage, France. Genotyping of the American samples was performed at the Broad Institute, USA.

The American and European GR cohorts were analysed separately and as a joint dataset. Data quality control was performed using the software package PLINK [41], removing SNPs and individuals with a call rate below 90%. Markers showing a low level of variability (MAF<0.01) were excluded from further association analysis. A total of 1,582 SNPs were removed due to platform-related genotyping inconsistencies due to differences in hybridization and calling algorithms used between two different sequencing platforms. Population stratification was estimated and visualized in multidimensional scaling plots (MDS) using PLINK to detect outliers and subgroups in the dataset after eliminating SNPs in high LD (r2>0.95). Due to the cryptic relatedness that often exists within a dog breed, the level of relatedness between individuals in each population was calculated using the GCTA software [42], and a genetic relationship matrix (grm) value of 0.25 was used as the cut-off threshold to remove highly related dogs (corresponding to half-sib level of relatedness). Regions associated with CMCT were detected by case-control genome-wide association analysis. PLINK and EMMAX software [41,43], were used to calculate association p-values, the latter software corrected for stratification and cryptic relatedness using mixed model statistics [43]. The LD-pruned SNP set was used for MDS, estimation of relatedness in GCTA and within the relationship matrix in EMMAX, whereas the full QC filtered SNP set was used for the association testing.

Quantile-quantile (QQ)-plots were created in R to assess possible genomic inflation and to establish suggestive significance levels. Permutation testing was performed in PLINK for the PLINK calculated association results, or GenABEL [44] for the mixed model association results. 10,000 permutations were performed. Minor allele frequencies and odds ratios, were calculated for each cohort (cases and controls) using PLINK.

Pair-wise r^2^–based LD between markers was used to evaluate the size of candidate regions and whether the associated loci were independent. The r^2^ calculations were performed using the Haploview and PLINK software packages [41,45]. Gene annotations were extracted from Ensemble [46] and UCSC [47].

### Targeted sequencing

Fifteen dogs (7 European (3 cases and 4 controls), and 8 American (5 cases and 3 controls)) were selected for sequencing of the associated genomic regions. A custom sequence capture array was designed (Nimblegen 2.1M solid array) to cover all associated regions. In total the capture array was designed to include 11.5 Mb DNA including the top associated regions CanFam2.0 cfa 20:41,149,999–43,000,000, cfa 20:46,099,999–49,700,000 and cfa14:14,599,999–15,450,000. Sequence capture was performed as previously described [48]. DNA from 15 individuals was individually barcoded and 3 DNA samples hybridized to each of 5 arrays. The DNA captured by each array was used to prepare a sequencing template library, and the libraries were sequenced on four Hi-Seq 2500 lanes.

Sequencing data was pre-processed and aligned using BWA [49], Samtools [50] and Picard to make bam files and to mark duplicate reads. Sequencing data was aligned to the CanFam 2.0 reference genome. Coverage of the targeted regions was 7-69x. GATK software [51] was used for data processing and genotype calling as well as filtering of variants. Called variants were annotated using SnpEff [52] and variants were scored according to conservation based on the 29 mammals data [53] using SEQScoring software [54], producing files which could be visualized graphically in the CanFam 2.0 UCSC browser. Bam files were visualized in IGV [55] to evaluate the presence of structural variants. Identified variants were evaluated in the CanFam3.1 genome assembly to assure that these variants were not due to faults in the CanFam2.0 assembly.

### Fine mapping of associated regions

SNPs that conformed to the haplotype for the most associated SNP were chosen for fine mapping. Priority was given to SNPs, which were conserved (as deemed by SEQscoring based on the 29 mammals data, including SNPs up to 6bp from conserved sites), coding SNPs, SNPs in UTR regions, SNPs upstream of genes in a predicted promoter region, and SNPs in introns. Additional SNPs, which did not conform to the risk haplotype, were chosen due to their location in interesting regions. SNPs were genotyped using the Sequenom MassArray iPLEX platform. Not all candidate SNPs could be genotyped due to iPLEX (multiPLEX) design limitations, or because of limitations in the number of SNPs that could be co-typed. Fine mapping data was analysed using Haploview, and 1,000,000 permutations were performed.

### RNA sequencing

Poly-A selected, strand-specific RNA sequencing was performed on a CMCT surgically excised from a GR. Sequencing libraries were prepared as described [56]. Normal marginal tissue was sampled as control. Samples were sequenced on one Illumina Hi-Seq 2500 lane. Data was analysed and aligned to CanFam3.1 using the tuxedo suite [21]. The sequence data was viewed in IGV.

### Immunohistochemistry

Immunohistochemistry was performed in order to visualize if hyaluronan is present in canine mast cell tumours. Slices of archived paraffin-embedded formalin-fixed CMCT tissue were dewaxed., and endogenous peroxidase blocked by incubation in 1% (v/v) H_2_0_2_ in 70% (v/v) ethanol for 5 min. Sections were washed sequentially in water and PBS and blocked for non-specific binding by a 30 min incubation in 1% (w/v) BSA in PBS. Sections were incubated over night at 4˚C with 2.5μg/ml Biotinylated Hyaluronan Binding Protein (AMS.HKD-BC41, AMSBIO) in 1% (w/v) BSA in PBS. Sections were washed with PBS and incubated with Vectastain Elite ABC Reagent (Vectastain Elite ABC Kit, Vector) for 30 minutes. After an additional wash in PBS the sections were incubated in diaminobenzidine for 7 min. The sections were rinsed in water and counterstained with 10% Mayer’s haematoxylin for 30s. The samples were then washed, dried for two minutes, and mounted in DPX mounting medium.

### Case selection for immunohistochemistry (IHC)

Eight cases of CMCT (6 dogs – 2 with multiple tumours), on which genome analysis was performed, were selected for IHC. In addition, four cases of CMCT in GRs were selected from AHT recent case submission and similarly stained.

As a negative control a portion of haired skin from the lateral chest of a dog with no evidence of skin disease was used.

Prior to IHC histopathological evaluation was performed on both test groups to confirm the presence of a CMCT. Blocks containing unaffected tissue margins were as far as possible selected for staining.

### Immunohistochemical evaluation

Medium to dark brown staining in a linear, granular or diffuse staining pattern in the epidermis, dermis, panniculus and in mast cells was considered as positive staining. A normal expected staining pattern as observed in the negative skin control included positive (linear) staining of the basement membrane of the epidermis, hair follicles, apocrine gland and blood vessels. Normal positive staining was also visible between dermal and pannicular collagen fibres and between adipocytes.

Positive staining in the mast cells was evaluated as nuclear (granular or diffuse), intracytoplasmic (granular or diffuse) or cytoplasmic membrane (linear). Positive staining was evaluated as light or intense.

### RNA extraction

RNA was extracted from RNAlater-preserved normal skin and CMCT samples using either TRIzol (Life Technologies) or the RNAeasy kit (QIAGEN). RNA integrity was evaluated by microfluidic electrophoresis (Agilent 2100 Bioanalyser RNA 6000 Nano Kit). cDNA was synthesised using the RT-for-PCR kit (Clontech).

### PCR validation of alternative splicing

PCR Primers were designed targeting the exon before and after the alternatively spliced exon. In addition, splice-specific primers were designed traversing the alternative splice site (see S6 Fig for design) as well as for the wild-type splice form as a control.

Alternative_splice_primer:

Forward: CATTGTCAAGCAGATGAAGATG,

Reverse: CTGCACACCG TTGTCAGCC

Splice_primer_control:

Forward: GACCCCTCCCGAGCGGATG

Reverse: As for alternative splice

Primer_traversing_alternative_spliced_exon

Forward: AGAGCACCATTGTCAAGCAG

Reverse: TCCGGATGACACAAGACAGATC

Quantitative PCR was performed on the 7900HT Fast Real-Time PCR (Applied Biosystems) using SyBr Green mastermix (Applied Biosystems). Delta Cq (Cq_normal_splice−_Cq_alternative_splice_) was calculated between the splice specific and alternative splice products for each cDNA sample. The PCR products were analysed by agarose gel analysis.

### Ethics statement

This study was approved by the Committee for Animal Care at the Massachusetts Institute of Technology, approval number MIT CAC 0910-074-13 and by the Uppsala Animal ethical board, approval number C2-12. No experimental animals were used in this research. Blood or buccal swaps were taken with owners consent. Tissue samples consisted of surplus material from surgical resections with owners consent.

## Supporting Information

S1 Fig Genotype frequencies for the most associated GWAS SNPs. Allele frequencies for US and European (EU) population are shown separately and combined (US/EU). A)Chr14: 14714009 G = risk, T = protective. B)Chr20: 42547825 C = risk, T = protective. C)Chr20: 48599799 A = risk, C = protective.
(PDF)

S2 Fig Sequence capture results and fine mapping of chromosome 14, 14Mb. The sequencing results of cfa14:14.6–15.0Mb viewed in USCS CanFam 2.0 after scoring using SEQscoring. Eight US dogs were sequenced with genotypes displayed as horizontal tracks. The genotype for the most associated SNP identified in the GWAS (cfa14: 14714009, blue arrow) is shown to the left (R/R = homozygous risk, R/r = heterozygous, r/r homozygous non-risk). The genotypes are colour-coded according to: yellow = homozygous reference allele, blue = homozygous opposite reference allele, green = heterozygous, red = homozygous evolutionary conserved SNPs, pink = heterozygous evolutionary conserved SNPs. The–log_10_(p) association p-values for the fine mapping are shown above as blue bars with the top SNP indicated by red arrow.
(PDF)

S3 Fig Sequence capture results and fine mapping chromosome 20 42Mb. The sequence capture results viewed in USCS CanFam 2.0 after scoring using SEQscoring. The region 41.1 Mb—42.2 Mb on chromosome 20 is shown. Sequence tracks for the 7 European and 8 United States samples are presented. The genotype for the most associated SNP identified in the GWAS (chr 20: 42547825) is shown to the left (R/R = homozygous risk, R/r = heterozygous, r/r homozygous non-risk). Red denotes conserved SNPs whilst blue denotes SNPs different from reference genome and green denotes heterozygotes. The yellow track denotes genotypes following the reference genotype. The–log10(p) association p-values for the combined fine mapping analysis for the United States and European population, as calculated by Haploview are shown above as blue bars.
(PDF)

S4 Fig Fine mapping plots showing p-values and LD structure. A) Plot showing the fine mapping results on chromosome 14 for the United States dogs only. LD structure shown in association to the most associated SNP in the area. B) Plot showing the fine mapping results on chromosome 20 42MB region for the United States and European dogs combined. LD structure shown in association to the most associated SNP in the area. C) Plot showing the fine mapping results on chromosome 20 42MB region for the United States and European dogs combined. LD structure shown in association to the second most associated SNP in the area. D) Plot showing the fine mapping results on chromosome 20 48MB region for the European dogs.LD structure shown in association to the most associated SNP in the area.
(PDF)

S5 Fig Genotype frequencies for the most associated fine mapping SNPs. Allele frequencies for US (US) and European (EU) population are shown separately and combined (US/EU). A) Chr20: 42080147 was found to be most associated in the European population, but was also associated in the United States population and hence the joint analysis improved the association. A = risk, G = protective. B) Chr 14: 14644897 was the most associated SNP in the United States population. This was an SNP identified as associated in the GWAS. No association to this SNP was found within the European population. C = risk, T = protective.
(PDF)

S6 Fig Primer design. Model showing the location of the splice specific primers used for detection of the alternative splicing of *GNAI2*. Primers specific for exon 3 skipping are shown in black. Primers specific for the wildtype isoform used as a control are shown in blue. Primers located on the exons around the alternatively spliced exon are shown in green (note this primer pair should give rise to two product in samples where alternative splicing occurs).
(PDF)

S7 Fig PCR products confirming the alternative splicing of *GNAI2*. A) PCR products from primers located in exon 2 and 4. Individuals carrying the A risk allele at chr 20:42080147, produce two products, both the normal and alternative isoforms. B) PCR products from splice specific primers traversing the normal splice site between exon 3 and 4 in *GNAI2*. Products seen in all samples, regardless of genotype. C) PCR products from primers traversing the alternative splice site, skipping exon 3. Products only seen in samples carrying the chr20:42080147 risk genotype A.
(PDF)

S8 Fig Model of the proposed consequences of the 42080147 SNP on the GNAI2 transcription and translation. The SNP chr 20: 420810147 alters the splice site in exon 3, introducing an alternatively spliced isoform of the *GNAI2* transcript. This alternatively spliced transcript is predicted to splice out of frame resulting in a truncated protein of 109 amino acids compared to the 355 amino acid wildtype protein.
(PDF)

S9 Fig Hyaluronan immunohistochemistry. Immunohistochemical staining of a intermediate grade mast cell tumour and normal control tissue using a Biotinylated Hyaluronic Acid Binding Protein to Stain for hyaluronan. Brown colour shows positive staining for hyaluronan. A) 50 x magnification image of intermediate grade cutaneous (grade 2) mast cell tumour from a golden retriever. Intense brown positive cytoplasmic membrane staining is seen surrounding the neoplastic mast cells (stained in blue) in the section. B) Same image as a) seen at 100x magnification (oil immersion). C) Normal cutaneous epidermis and panniculus tissue from a golden Retriever seen at 20x magnification. Light brown staining of collagen is seen in the dermal layer. D) Same image as c) at 50 x magnification. Intense brown staining is seen in the epidermal basement membrane, which is known to contain large amounts of hyaluronan. Adjacent collagen showed variable light brown staining. E) Staining of adipose control tissue (panniculus) 50 x magnification showing light positive staining of extracellular matrix between adipose cells.
(PNG)

S1 Table A) Table listing the associated SNPs from the United States GWAS analysis. B) Table listing the associated SNPs from the European GWAS analysis. A1 = risk allele. A2 = non risk allele. F_A = A1 allele frequency for affected individuals, F_U = A1 allele frequency for unaffected individuals.
(PDF)

S2 Table Table showing the most associated SNPs from the US fine mapping analysis. Allele frequencies for cases and controls. p-values and permutated p-values (1.000.000 permutations) are shown. P-values for SNPs, which were included in the GWAS and repeated in the fine mapping are shown. Only the 20 most associated iplex SNPs are shown.
(PDF)

S3 Table Table showing all 97 iplex SNPs used in the analysis. Allele frequencies. p-values and permutated p-values (1.000.000 permutations) are showing for the European (EU) analysis and the combined European and United States analysis. GWAS p-values for SNPs, which were included in the GWAS and repeated in the iplex are shown.
(PDF)
