# Supplementary figures and images for: Genome-Wide Association Study of Golden Retrievers Identifies Germ-Line Risk Factors Predisposing to Mast Cell Tumours
Source: PLoS Genet. 2015 Nov 20;11(11):e1005647. doi: 10.1371/journal.pgen.1005647 (PMC4654484; doi:10.1371/journal.pgen.1005647)

# Supp. Figure 1

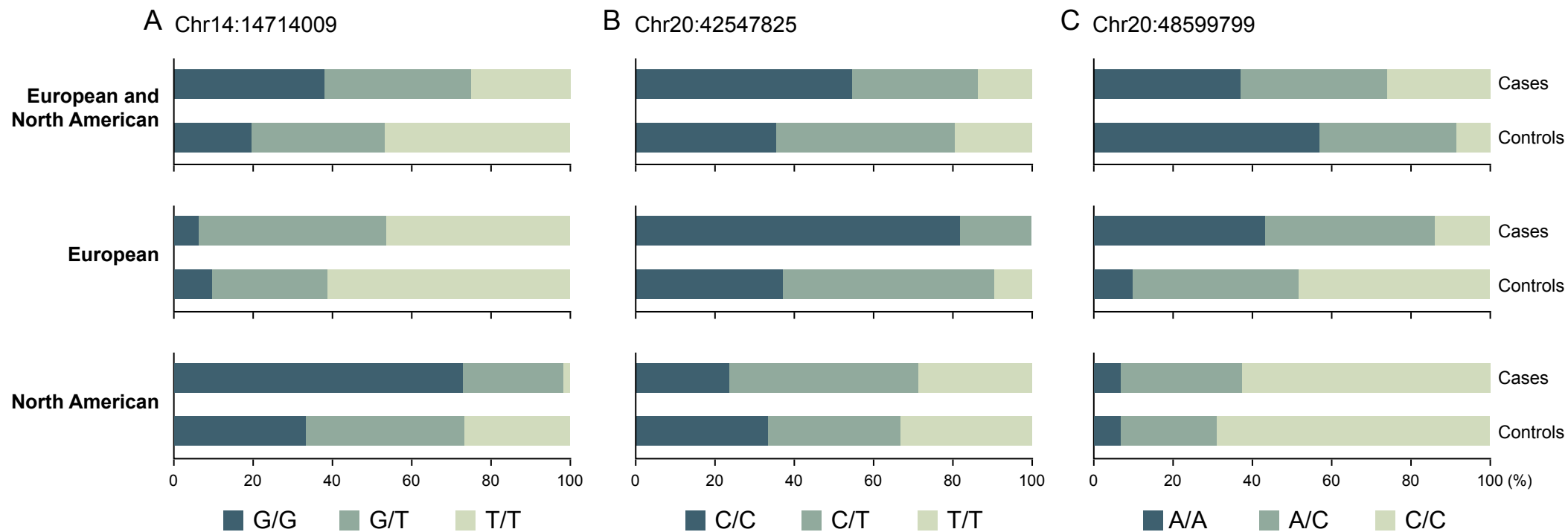

Supplement: S1 Fig — Allele frequencies for US and European (EU) population are shown separately and combined (US/EU). A)Chr14: 14714009 G = risk, T = protective. B)Chr20: 42547825 C = risk, T = protective. C)Chr20: 48599799 A = risk, C = protective. (PDF) [file pgen.1005647.s001.pdf]

## Supp. Figure 2

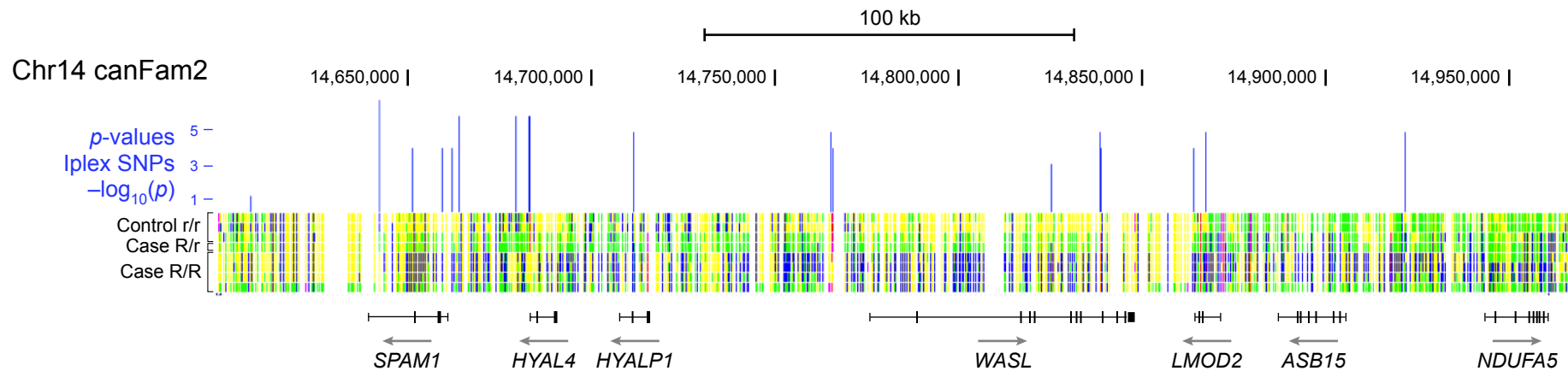

Supplement: S2 Fig — The sequencing results of cfa14:14.6–15.0Mb viewed in USCS CanFam 2.0 after scoring using SEQscoring. Eight US dogs were sequenced with genotypes displayed as horizontal tracks. The genotype for the most associated SNP identified in the GWAS (cfa14: 14714009, blue arrow) is shown to the left (R/R = homozygous risk, R/r = heterozygous, r/r homozygous non-risk). The genotypes are colour-coded according to: yellow = homozygous reference allele, blue = homozygous opposite reference allele, green = heterozygous, red = homozygous evolutionary conserved SNPs, pink = heterozygous evolutionary conserved SNPs. The–log10(p) association p-values for the fine mapping are shown above as blue bars with the top SNP indicated by red arrow. (PDF) [file pgen.1005647.s002.pdf]

# Supp. Figure 3

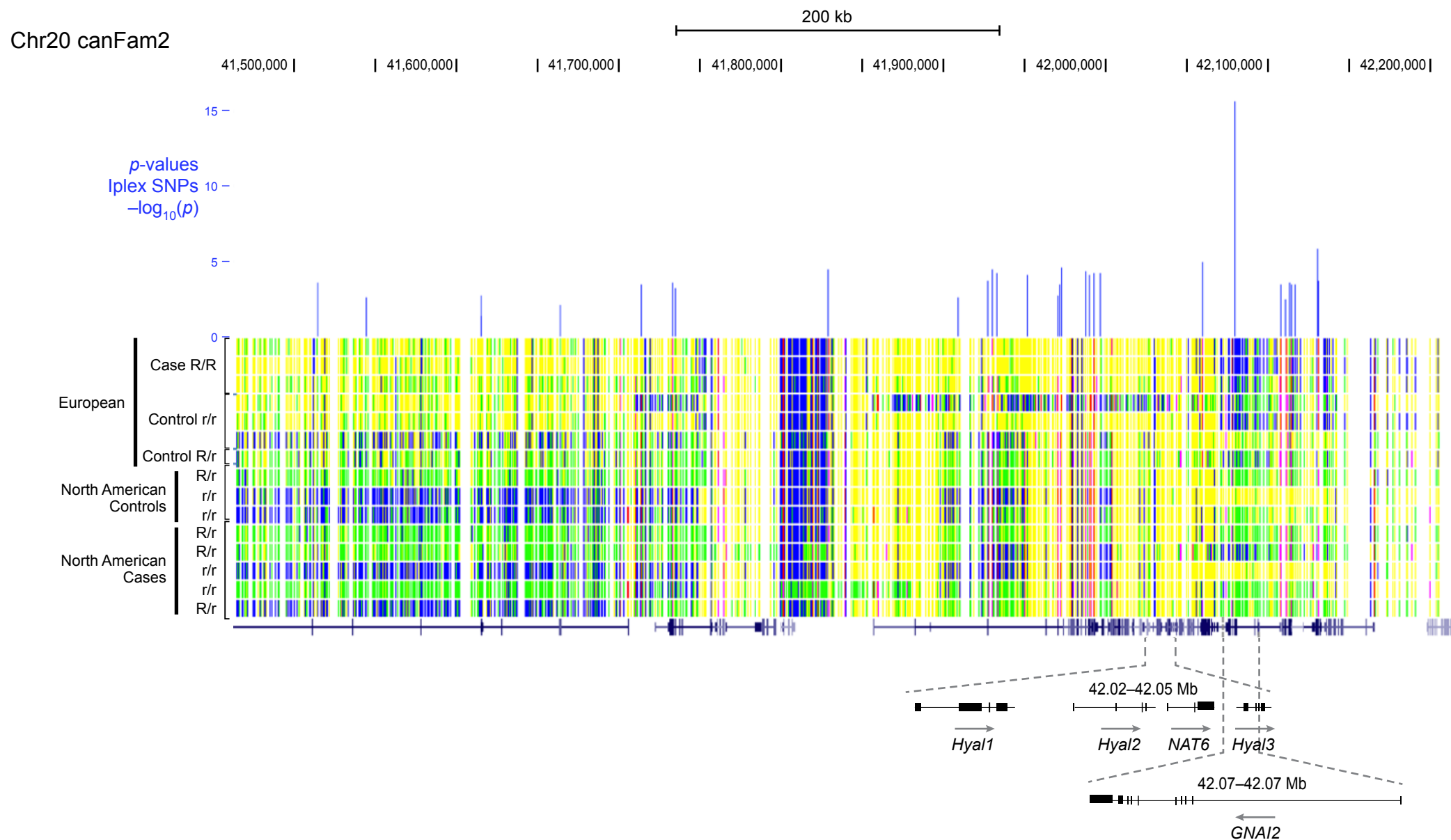

Supplement: S3 Fig — The sequence capture results viewed in USCS CanFam 2.0 after scoring using SEQscoring. The region 41.1 Mb—42.2 Mb on chromosome 20 is shown. Sequence tracks for the 7 European and 8 United States samples are presented. The genotype for the most associated SNP identified in the GWAS (chr 20: 42547825) is shown to the left (R/R = homozygous risk, R/r = heterozygous, r/r homozygous non-risk). Red denotes conserved SNPs whilst blue denotes SNPs different from reference genome and green denotes heterozygotes. The yellow track denotes genotypes following the reference genotype. The–log10(p) association p-values for the combined fine mapping analysis for the United States and European population, as calculated by Haploview are shown above as blue bars. (PDF) [file pgen.1005647.s003.pdf]

# Supp. Figure 4

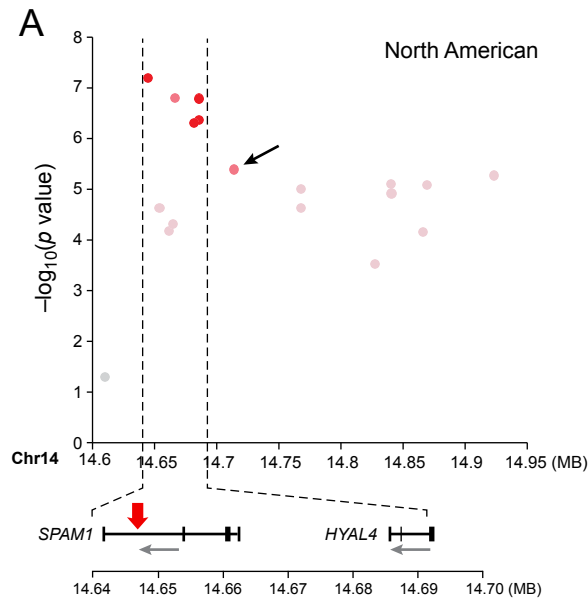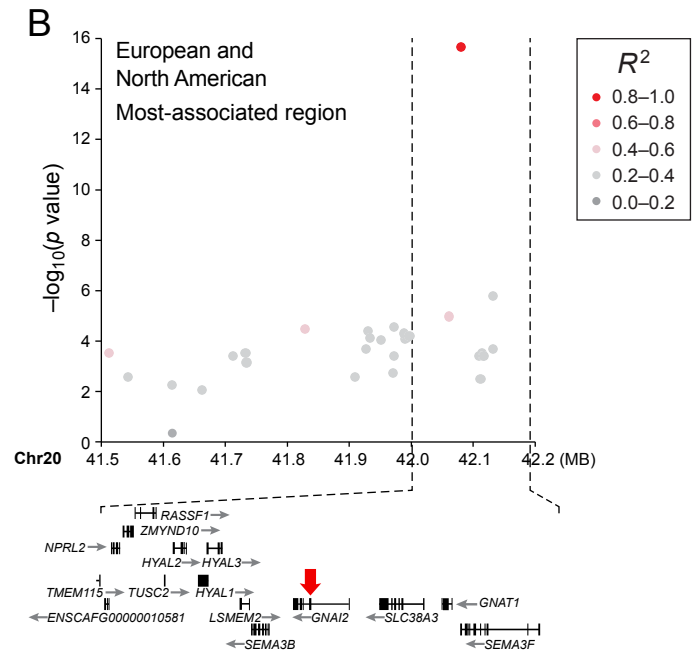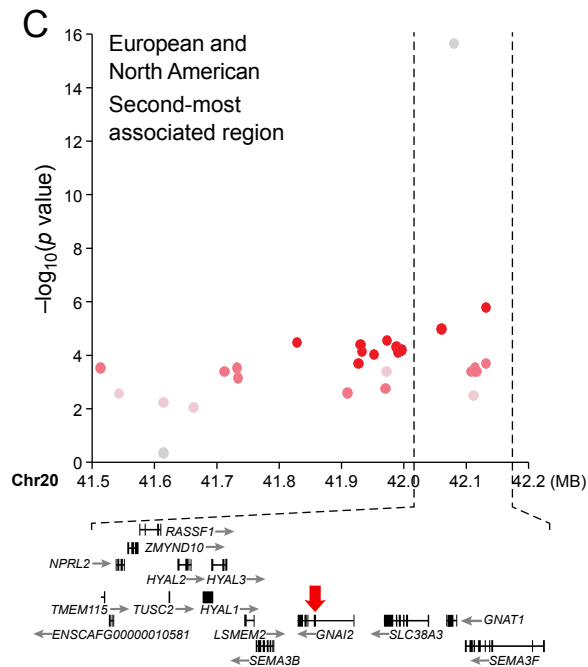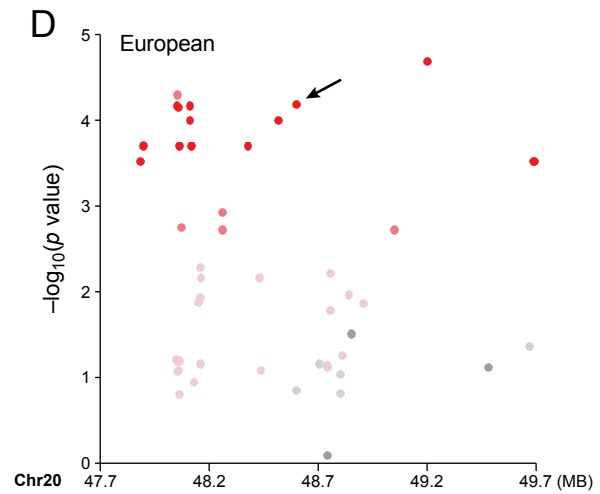

Supplement: S4 Fig — A) Plot showing the fine mapping results on chromosome 14 for the United States dogs only. LD structure shown in association to the most associated SNP in the area. B) Plot showing the fine mapping results on chromosome 20 42MB region for the United States and European dogs combined. LD structure shown in association to the most associated SNP in the area. C) Plot showing the fine mapping results on chromosome 20 42MB region for the United States and European dogs combined. LD structure shown in association to the second most associated SNP in the area. D) Plot showing the fine mapping results on chromosome 20 48MB region for the European dogs.LD structure shown in association to the most associated SNP in the area. (PDF) [file pgen.1005647.s004.pdf]

# Supp. Figure 5

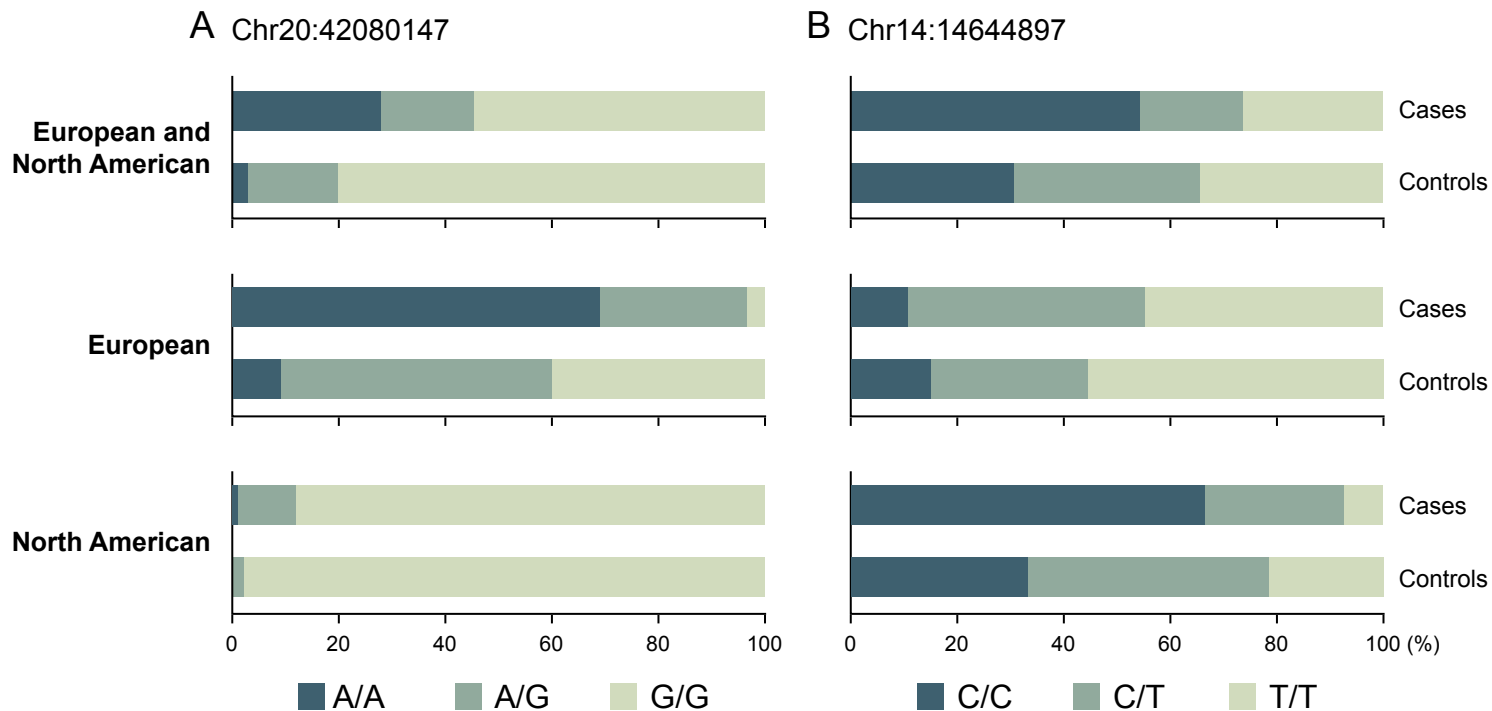

Supplement: S5 Fig — Allele frequencies for US (US) and European (EU) population are shown separately and combined (US/EU). A) Chr20: 42080147 was found to be most associated in the European population, but was also associated in the United States population and hence the joint analysis improved the association. A = risk, G = protective. B) Chr 14: 14644897 was the most associated SNP in the United States population. This was an SNP identified as associated in the GWAS. No association to this SNP was found within the European population. C = risk, T = protective. (PDF) [file pgen.1005647.s005.pdf]

## Supp. Figure 6

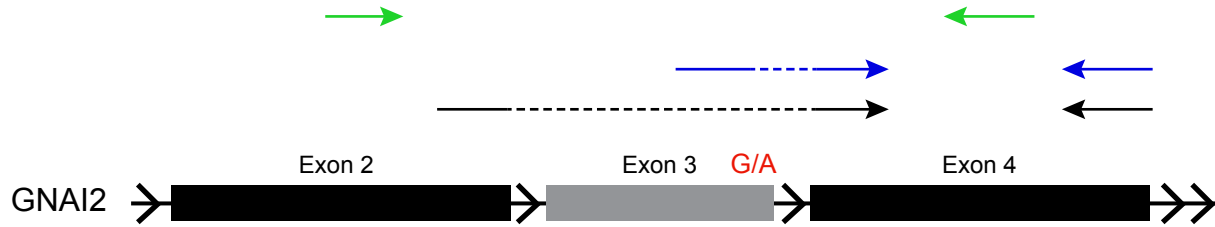

Supplement: S6 Fig — Model showing the location of the splice specific primers used for detection of the alternative splicing of GNAI2. Primers specific for exon 3 skipping are shown in black. Primers specific for the wildtype isoform used as a control are shown in blue. Primers located on the exons around the alternatively spliced exon are shown in green (note this primer pair should give rise to two product in samples where alternative splicing occurs). (PDF) [file pgen.1005647.s006.pdf]

## Supp. Figure 8

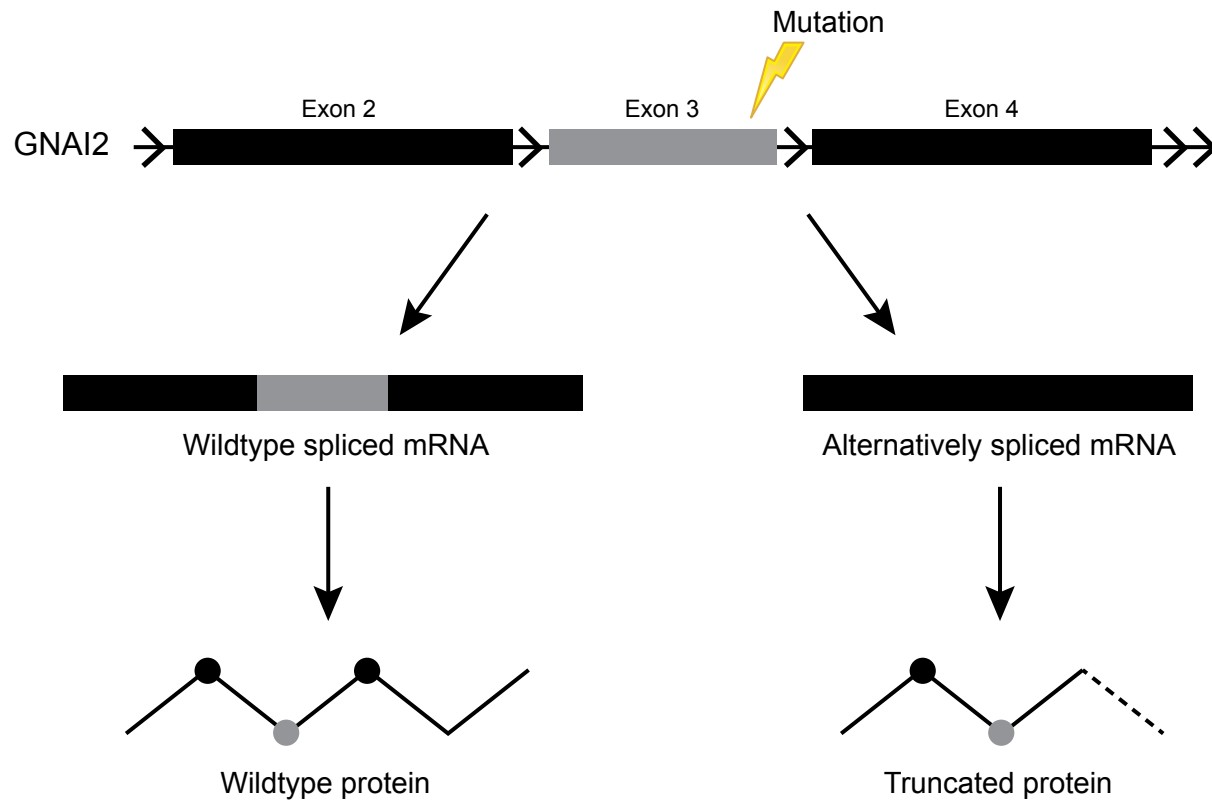

Supplement: S8 Fig — The SNP chr 20: 420810147 alters the splice site in exon 3, introducing an alternatively spliced isoform of the GNAI2 transcript. This alternatively spliced transcript is predicted to splice out of frame resulting in a truncated protein of 109 amino acids compared to the 355 amino acid wildtype protein. (PDF) [file pgen.1005647.s008.pdf]

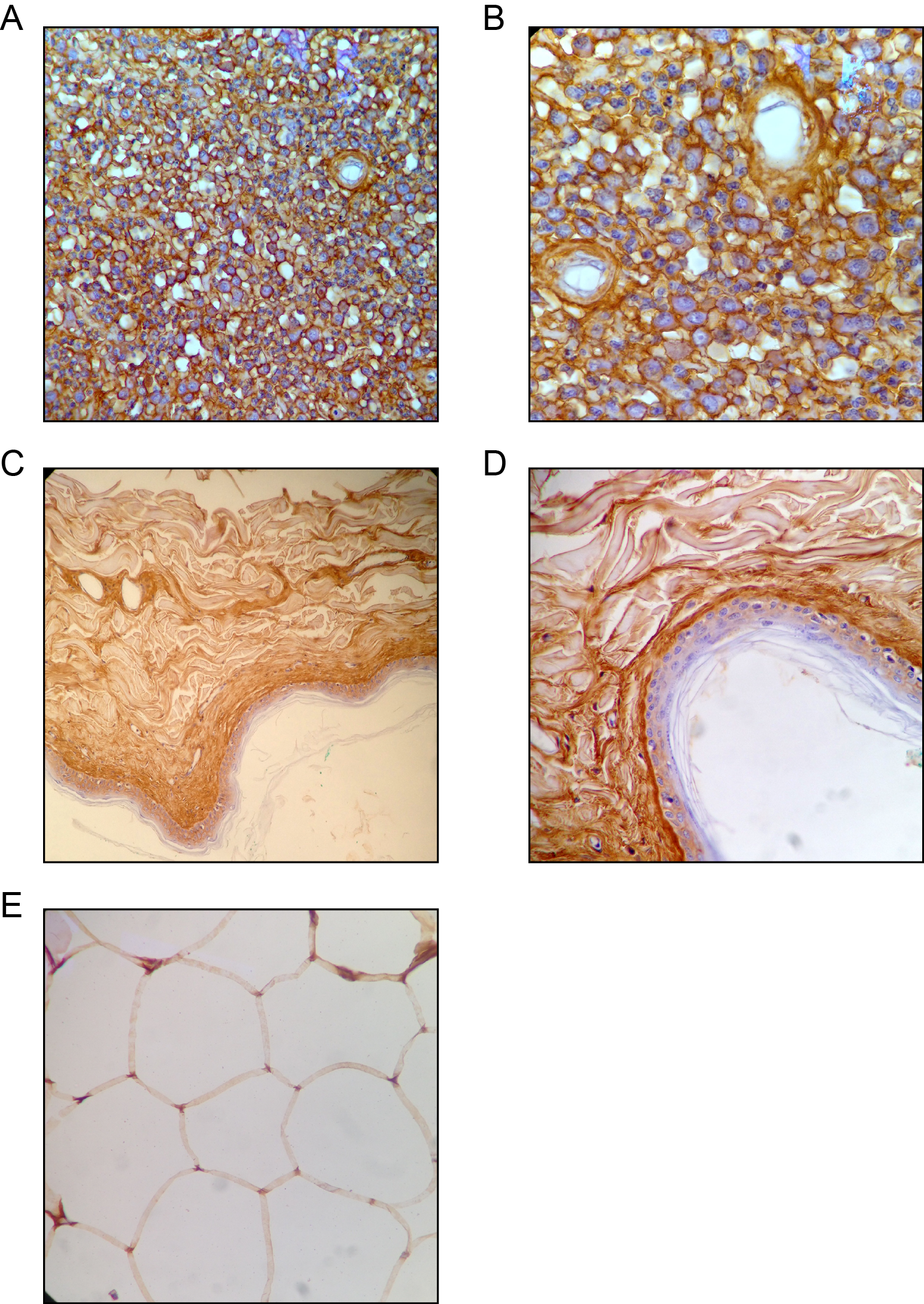

Supplement: S9 Fig — Immunohistochemical staining of a intermediate grade mast cell tumour and normal control tissue using a Biotinylated Hyaluronic Acid Binding Protein to Stain for hyaluronan. Brown colour shows positive staining for hyaluronan. A) 50 x magnification image of intermediate grade cutaneous (grade 2) mast cell tumour from a golden retriever. Intense brown positive cytoplasmic membrane staining is seen surrounding the neoplastic mast cells (stained in blue) in the section. B) Same image as a) seen at 100x magnification (oil immersion). C) Normal cutaneous epidermis and panniculus tissue from a golden Retriever seen at 20x magnification. Light brown staining of collagen is seen in the dermal layer. D) Same image as c) at 50 x magnification. Intense brown staining is seen in the epidermal basement membrane, which is known to contain large amounts of hyaluronan. Adjacent collagen showed variable light brown staining. E) Staining of adipose control tissue (panniculus) 50 x magnification showing light positive staining of extracellular matrix between adipose cells. (PNG) [file pgen.1005647.s009.png]
