# Supplementary material for: Genome-Wide Association Study of Golden Retrievers Identifies Germ-Line Risk Factors Predisposing to Mast Cell Tumours
Source: PLoS Genet. 2015 Nov 20;11(11):e1005647. doi: 10.1371/journal.pgen.1005647 (PMC4654484; doi:10.1371/journal.pgen.1005647)
Supplement: S7 Fig — A) PCR products from primers located in exon 2 and 4. Individuals carrying the A risk allele at chr 20:42080147, produce two products, both the normal and alternative isoforms. B) PCR products from splice specific primers traversing the normal splice site between exon 3 and 4 in GNAI2. Products seen in all samples, regardless of genotype. C) PCR products from primers traversing the alternative splice site, skipping exon 3. Products only seen in samples carrying the chr20:42080147 risk genotype A. (PDF) [file pgen.1005647.s007.pdf]

Supp. Figure 7

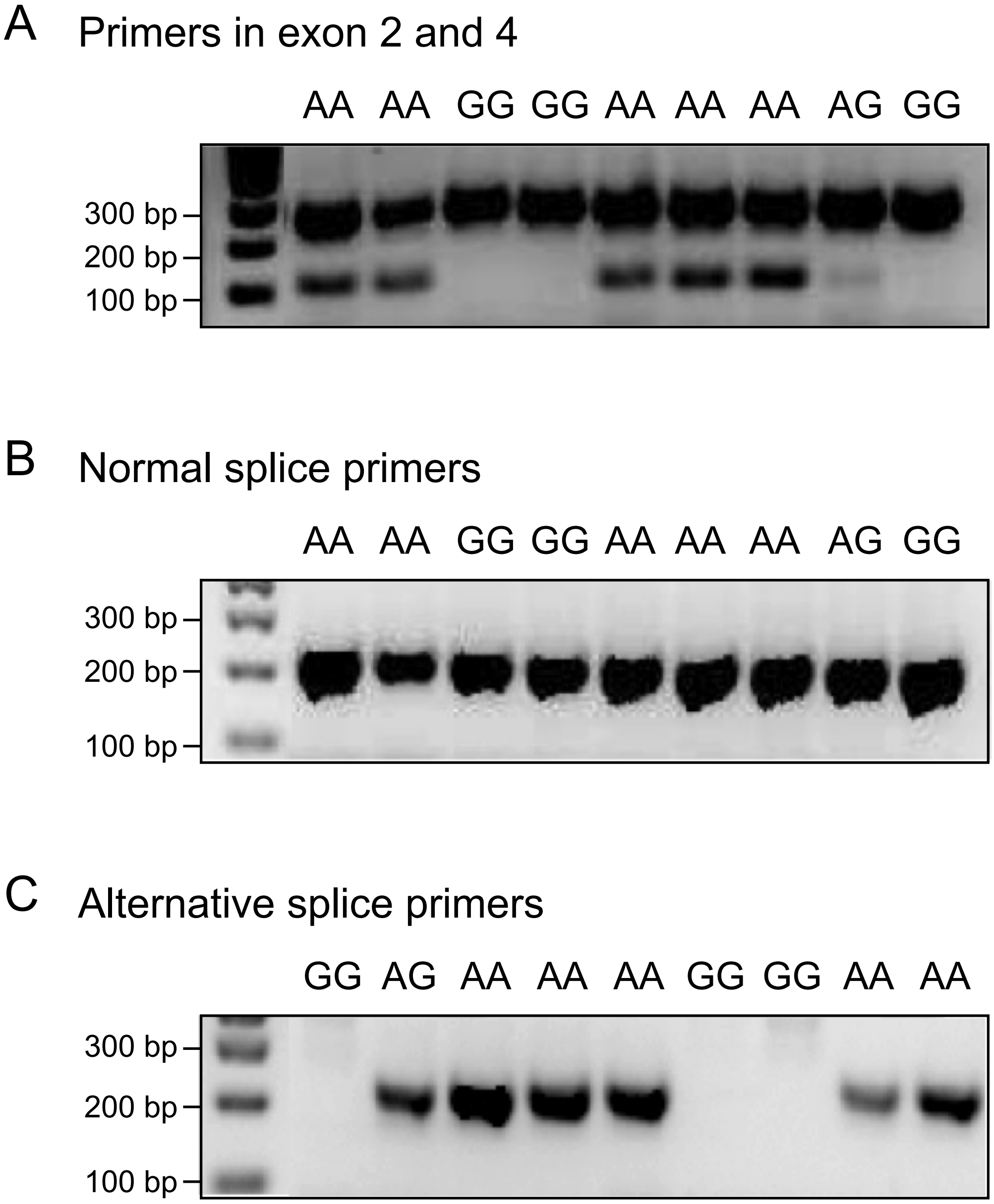

AA = homozygous risk

GG = homozygous non-risk

AG = heterozygote
