# Supplementary material for: Genome-Wide Association Study of Golden Retrievers Identifies Germ-Line Risk Factors Predisposing to Mast Cell Tumours
Source: PLoS Genet. 2015 Nov 20;11(11):e1005647. doi: 10.1371/journal.pgen.1005647 (PMC4654484; doi:10.1371/journal.pgen.1005647)
Supplement: S1 Table — A1 = risk allele. A2 = non risk allele. F_A = A1 allele frequency for affected individuals, F_U = A1 allele frequency for unaffected individuals. (PDF) [file pgen.1005647.s010.pdf]

CHR	SNP	POSITION	A1	A2	F_A	F_U	P
14	BICF2P867665	14714009	G	T	0.86	0.53	3.16E-07
14	BICF2G630521572	14670361	T	C	0.77	0.44	1.33E-06
14	BICF2G630521696	14756089	G	A	0.77	0.44	1.33E-06
14	TIGRP2P186605	14727905	G	A	0.76	0.44	2.63E-06
14	BICF2G630521678	14740313	A	G	0.76	0.44	2.63E-06
14	BICF2G630521681	14743663	C	T	0.76	0.44	2.63E-06
14	BICF2G630521558	14644897	C	T	0.85	0.56	3.30E-06
14	BICF2G630521619	14685543	C	T	0.85	0.56	3.30E-06
14	BICF2G630521606	14682089	T	C	0.84	0.56	7.03E-06

S1a table)

CHR	SNP	BP	A1	A2	F_A	F_U	P
20	BICF2P301921	48599799	A	C	0.6462	0.3065	4.63E-07
20	BICF2P623297	49201505	G	A	0.6308	0.2903	8.07E-07
20	BICF2P299292	48377580	A	C	0.6462	0.3145	1.20E-06
20	BICF2S22934685	42547825	C	T	0.91538	0.6371	1.39E-06
20	BICF2P1444805	42957449	A	G	0.933846	0.7016	2.58E-06
20	BICF2S23324924	42988068	T	C	0.93846	0.7016	2.58E-06
20	BICF2P1465662	48963283	C	T	0.6385	0.3145	2.97E-06
20	BICF2P1050738	47970548	C	T	0.6462	0.3197	2.99E-06
20	BICF2P1084749	47963302	G	A	0.6462	0.3226	3.09E-06
20	BICF2P951309	47944650	C	A	0.6462	0.3226	3.09E-06
20	BICF2P20683	41576457	G	A	0.93846	0.7083	4.22E-06
20	BICF2P1185290	42004062	C	T	0.93077	0.6967	4.86E-06
20	BICF2P271393	41745091	G	A	0.92308	0.6694	5.79E-06
20	BICF2P372450	41734129	A	G	0.92308	0.6694	5.79E-06
20	BICF2P453555	41709258	C	T	0.92308	0.6694	5.79E-06
20	BICF2P1324128	47908830	G	C	0.6308	0.3226	6.25E-06
20	BICF2P1241961	42114184	G	A	0.93846	0.7097	6.30E-06
20	BICF2P250980	42095538	G	A	0.93846	0.7097	6.30E-06
20	BICF2S23160763	42071038	T	C	0.93846	0.7097	6.30E-06
20	BICF2P549	41466952	G	A	0.93846	0.7131	6.39E-06
20	BICF2P1405309	48077227	C	T	0.6538	0.3468	6.66E-06
20	BICF2P112281	41991115	A	G	0.93077	0.7016	7.97E-06
20	BICF2P1231294	41951828	T	C	0.93077	0.7016	7.97E-06
20	BICF2P1310301	41927031	G	A	0.93077	0.7016	7.97E-06
20	BICF2P1310305	41930509	G	A	0.93077	0.7016	7.97E-06
20	BICF2P304809	41924733	C	T	0.93077	0.7016	7.97E-06
20	BICF2P327134	41516957	A	C	0.93077	0.7016	7.97E-06
20	BICF2P541405	41954052	C	A	0.93077	0.7016	7.97E-06
20	BICF2P854185	41916205	G	A	0.93077	0.7016	7.97E-06
20	BICF2S23030593	49051702	C	T	0.6385	0.3306	8.96E-06
20	BICF2P1139808	41395277	T	C	0.93846	0.7177	9.91E-06
20	BICF2P116133	41241178	G	A	0.93846	0.7177	9.91E-06
20	BICF2P1163972	41618769	C	A	0.93846	0.7177	9.91E-06
20	BICF2P1224909	41337123	G	A	0.93846	0.7177	9.91E-06
20	BICF2P1314689	41215117	A	C	0.93846	0.7177	9.91E-06
20	BICF2P1342476	41411067	A	G	0.93846	0.7177	9.91E-06
20	BICF2P1517463	41697094	C	G	0.93846	0.7177	9.91E-06
20	BICF2P257870	41488878	A	G	0.93846	0.7177	9.91E-06
20	BICF2P360884	41586182	T	C	0.93846	0.7177	9.91E-06
20	BICF2P408113	41229381	G	T	0.93846	0.7177	9.91E-06
20	BICF2P413074	41345712	A	G	0.93846	0.7177	9.91E-06
20	BICF2P471574	41291981	C	T	0.93846	0.7177	9.91E-06
20	BICF2P509577	41310875	C	A	0.93846	0.7177	9.91E-06
20	BICF2P626859	41365616	A	G	0.93846	0.7177	9.91E-06

20	BICF2P648601	41424761	A	G	0.93846	0.7177	9.91E-06
20	BICF2P687775	41662902	A	G	0.93846	0.7177	9.91E-06
20	BICF2P716239	41900414	G	A	0.93846	0.7177	9.91E-06
20	BICF2P735611	41327714	G	A	0.93846	0.7177	9.91E-06
20	BICF2P769104	41422308	T	C	0.93846	0.7177	9.91E-06
20	BICF2P789266	41454760	A	G	0.93846	0.7177	9.91E-06
20	BICF2P914653	41217592	T	C	0.93846	0.7177	9.91E-06
20	BICF2P968727	41387018	T	C	0.93846	0.7177	9.91E-06
20	BICF2S23114565	41304489	A	G	0.93846	0.7177	9.91E-06
20	BICF2S23351441	41493229	A	C	0.93846	0.7177	9.91E-06
20	TIGRP2P274855	41180269	G	A	0.93846	0.7177	9.91E-06
20	TIGRP2P274858	41271157	G	T	0.93846	0.7177	9.91E-06
20	TIGRP2P274899	41795286	C	T	0.93846	0.7177	9.91E-06
20	BICF2P766049	49690415	A	G	0.6231	0.3145	1.28E-05
20	BICF2P983977	41642791	T	C	0.93846	0.7213	1.69E-05
20	BICF2P302160	48837386	C	A	0.6769	0.4032	2.23E-05
20	BICF2S23532900	47839318	G	T	0.6385	0.3548	2.60E-05
29	BICF2P428532	6430664	T	C	0.91538	0.7339	2.77E-05
29	BICF2P435646	6396608	A	G	0.91538	0.7339	2.77E-05
29	BICF2P876590	6409890	G	A	0.91538	0.7339	2.77E-05
20	BICF2S2305218	42975776	G	A	0.95312	0.7459	3.84E-05
20	BICF2S2295117	42587791	A	G	0.92308	0.6774	4.09E-05
20	BICF2P508868	41723260	G	C	0.93846	0.7288	4.12E-05
20	BICF2S2376197	49726685	C	T	0.6308	0.3468	4.82E-05
29	BICF2P775371	6600700	G	A	0.91538	0.7419	4.99E-05
29	BICF2P969352	6484242	C	T	0.90769	0.7258	5.63E-05
29	BICF2P995575	6501127	C	T	0.90769	0.7258	5.63E-05
29	BICF2S23152485	6515084	C	A	0.90769	0.7258	5.63E-05
20	BICF2S23333987	36006050	A	T	0.8077	0.5164	5.89E-05
20	BICF2P878447	47709032	C	T	0.6385	0.3629	6.44E-05
20	BICF2P458881	42477560	T	C	0.91538	0.6613	7.38E-05
20	BICF2P861824	42483020	T	C	0.91538	0.6613	7.38E-05
20	BICF2P1313482	47607715	A	G	0.6308	0.3548	8.01E-05
20	BICF2P1429559	47588306	T	A	0.6308	0.3548	8.01E-05
20	BICF2P1429562	47585373	C	T	0.6308	0.3548	8.01E-05
20	BICF2P787087	47551706	A	G	0.6308	0.3548	8.01E-05
20	BICF2P696014	46174459	A	T	0.6154	0.3145	8.48E-05
20	BICF2P716231	46238879	T	G	0.6154	0.3145	8.48E-05
20	BICF2P81421	46187197	A	G	0.6154	0.3145	8.48E-05
1	BICF2P1150815	112874215	A	T	0.6935	0.4839	9.10E-05
29	BICF2P1061684	6489580	A	G	0.90769	0.7339	1.00E-04
29	BICF2P716492	6459761	C	T	0.90769	0.7339	1.00E-04
20	BICF2P1078264	36638018	C	T	0.7615	0.4435	1.07E-04
20	BICF2S23432636	36319043	A	C	0.7615	0.4435	1.07E-04
20	BICF2S2343757	36431095	T	C	0.7615	0.4435	1.07E-04

20	BICF2S2355724	36435937	G	T	0.7615	0.4435	1.07E-04
20	BICF2S23725316	46197200	C	T	0.6231	0.3306	1.13E-04
34	BICF2P388344	22273101	A	G	0.6615	0.4113	1.18E-04
1	BICF2P1005385	112880986	C	T	0.6846	0.4839	1.28E-04
1	BICF2P119057	112909660	G	A	0.6846	0.4839	1.28E-04
1	BICF2P61512	112887495	C	T	0.6846	0.4839	1.28E-04
1	BICF2S23522133	112857704	G	T	0.6846	0.4833	1.45E-04
20	BICF2P134412	42151061	T	C	0.93846	0.7321	1.46E-04
20	BICF2P1173489	42415710	G	A	0.94615	0.75	1.46E-04
20	BICF2P476394	42406453	T	C	0.94615	0.75	1.46E-04
20	BICF2P611903	42083608	C	G	0.94615	0.75	1.46E-04
20	BICF2S23139889	42936673	C	T	0.94615	0.75	1.46E-04
20	BICF2G630448341	53017458	C	T	0.5308	0.2787	1.50E-04
20	BICF2S23510370	48264265	G	A	0.6846	0.4274	1.61E-04
34	BICF2P975957	22369974	C	T	0.6538	0.4032	1.85E-04
20	BICF2S23427242	47068232	A	G	0.6231	0.3548	1.90E-04
20	BICF2P1110958	37772947	A	G	0.7538	0.4677	1.91E-04
20	BICF2P1191632	42272764	G	A	0.93846	0.7339	2.15E-04
20	BICF2P927225	42375806	T	C	0.93846	0.7339	2.15E-04
20	TIGRP2P274941	42386452	T	C	0.93846	0.7339	2.15E-04
20	BICF2S23340206	44955843	C	A	0.8385	0.5968	2.29E-04
20	BICF2S23713080	44941862	C	A	0.8385	0.5968	2.29E-04
20	BICF2S233350	45467889	T	C	0.8231	0.541	2.38E-04
20	G1102F25S86	36081820	T	C	0.7538	0.4516	2.45E-04
20	BICF2S2309267	36310170	A	G	0.8231	0.5484	2.55E-04
20	BICF2P1144529	47520654	T	C	0.6308	0.3596	2.82E-04
20	TIGRP2P274298	38744377	G	A	0.8154	0.5726	3.03E-04
20	BICF2P294403	46448776	A	G	0.6308	0.3417	3.22E-04
20	BICF2P1317092	46438016	A	G	0.6308	0.3468	3.31E-04
20	BICF2P1179081	45301965	T	A	0.8308	0.5726	3.35E-04
20	BICF2P608559	45311886	A	G	0.8308	0.5726	3.35E-04
20	BICF2P782456	45327022	T	C	0.8308	0.5726	3.35E-04
34	BICF2P113954	22669214	C	T	0.3538	0.129	3.37E-04
34	BICF2P1297385	22649562	A	G	0.3538	0.129	3.37E-04
34	TIGRP2P395985	22680347	T	C	0.3538	0.129	3.37E-04
11	BICF2P1304130	9163839	G	A	0.8846	0.7581	3.45E-04
20	BICF2P88083	39777883	G	A	0.8385	0.5968	3.48E-04
20	BICF2P299210	45359331	G	T	0.8308	0.5806	3.67E-04
20	BICF2P911789	45335884	G	A	0.8308	0.5806	3.67E-04
20	BICF2P926434	45355933	A	G	0.8308	0.5806	3.67E-04
2	BICF2P221622	25975450	C	G	0.2385	0.08065	3.70E-04
34	BICF2S23058860	22727710	T	C	0.3462	0.121	3.74E-04
2	BICF2S23028839	29377632	A	G	0.8308	0.629	4.16E-04
34	BICF2P800727	22415237	G	A	0.4692	0.2581	4.18E-04
11	BICF2P734487	4851446	A	G	0.8077	0.621	4.31E-04

33	BICF2G630248964	12744414	C	A	0.98462	0.8871	4.35E-04
33	BICF2G630248967	12744897	T	G	0.98462	0.8871	4.35E-04
34	BICF2P844581	22407053	G	A	0.4615	0.25	4.50E-04
34	BICF2P1019737	23454842	A	T	0.5077	0.2419	4.51E-04
34	BICF2P1307375	23447846	G	A	0.5077	0.2419	4.51E-04
34	BICF2S23641565	23462830	G	A	0.5077	0.2419	4.51E-04
34	TIGRP2P396123	23465333	G	A	0.5077	0.2419	4.51E-04
34	BICF2P580443	23472767	G	A	0.4923	0.2258	4.55E-04
29	BICF2G630621992	5036348	C	T	0.992308	0.879	4.69E-04
10	BICF2P1383492	25657545	C	G	0.4846	0.2661	4.89E-04
10	BICF2P418144	25663694	T	C	0.4846	0.2661	4.89E-04
10	BICF2P549512	25635053	C	T	0.4846	0.2661	4.89E-04
34	TIGRP2P395933	22360003	G	A	0.5	0.2787	4.92E-04
20	BICF2P560295	39815670	T	C	0.8385	0.6048	5.02E-04
20	BICF2P619863	39803010	T	C	0.8385	0.6048	5.02E-04
20	BICF2S23447001	39787259	G	A	0.8385	0.6048	5.02E-04
20	BICF2S23448192	39794609	G	A	0.8385	0.6048	5.02E-04
20	BICF2G630448354	53010725	T	C	0.5385	0.3065	5.21E-04
20	BICF2P800294	48867002	T	C	0.7308	0.4758	5.31E-04
34	BICF2P1355086	41206536	C	T	0.7692	0.90323	5.33E-04
36	BICF2S23038776	28970225	A	C	0.8923	0.7016	5.36E-04
2	BICF2P293793	25589556	C	T	0.2462	0.09677	5.42E-04
2	BICF2P330215	25586872	T	C	0.2462	0.09677	5.42E-04
34	BICF2S23158791	22182558	T	G	0.3385	0.121	5.48E-04
20	BICF2S22923756	44198701	C	T	0.8385	0.5887	5.52E-04
20	BICF2S22952333	44027026	A	G	0.8385	0.5887	5.52E-04
20	BICF2S23150491	44312048	G	A	0.8385	0.5887	5.52E-04
20	BICF2S23152344	44167432	C	T	0.8385	0.5887	5.52E-04
20	BICF2S23158681	43941778	A	G	0.8385	0.5887	5.52E-04
20	BICF2S23212666	44128697	T	C	0.8385	0.5887	5.52E-04
20	BICF2S23216159	44105651	A	G	0.8385	0.5887	5.52E-04
20	BICF2S23334554	43935688	A	G	0.8385	0.5887	5.52E-04
20	BICF2S23343399	44122748	C	T	0.8385	0.5887	5.52E-04
20	BICF2S23415717	44354720	C	T	0.8385	0.5887	5.52E-04
20	BICF2S23726023	44246884	T	C	0.8385	0.5887	5.52E-04
20	BICF2S23748153	44331745	A	G	0.8385	0.5887	5.52E-04
20	BICF2P1202229	49028407	C	T	0.7308	0.4758	5.69E-04
34	BICF2S23721551	22786544	G	A	0.3692	0.1583	5.75E-04
1	BICF2P1393892	110051203	T	C	0.7385	0.5323	6.05E-04
1	BICF2P772339	110101150	A	G	0.7385	0.5323	6.05E-04
1	BICF2P949608	110067267	C	A	0.7385	0.5323	6.05E-04
2	BICF2S23128513	25605276	A	G	0.2385	0.08871	6.07E-04
34	BICF2G630461610	22576344	T	A	0.3769	0.1694	6.07E-04
36	BICF2G630758305	29213130	T	A	0.9	0.7339	6.38E-04
10	BICF2P1221052	25673093	C	A	0.4846	0.2742	6.44E-04

10	BICF2P1383491	25657039	C	G	0.4846	0.2742	6.44E-04
16	BICF2G630816181	51503730	C	A	0.3	0.123	6.47E-04
29	BICF2P774201	7177794	T	G	0.98462	0.879	6.51E-04
2	BICF2P1292637	25567596	A	G	0.2385	0.08871	6.83E-04
2	BICF2P1426477	25614300	G	C	0.2385	0.08871	6.83E-04
20	BICF2S23549218	38864849	G	C	0.8462	0.6371	6.93E-04
20	BICF2S23418753	39230593	C	T	0.8462	0.621	6.96E-04
20	BICF2S23763114	44001043	G	A	0.8385	0.5902	7.19E-04
2	BICF2P1055079	42016199	C	T	0.8077	0.5726	7.31E-04
29	BICF2S23216164	31110727	A	C	0.6077	0.4032	7.32E-04
1	BICF2P357751	110078102	G	T	0.7385	0.5328	7.44E-04
6	BICF2S23449711	64796234	T	C	0.6538	0.4113	7.45E-04
20	BICF2P861196	44849564	T	C	0.8308	0.6048	7.46E-04
20	BICF2P1242966	39365169	C	T	0.8462	0.629	7.54E-04
20	BICF2S23450151	39397583	A	C	0.8462	0.629	7.54E-04
20	BICF2S22931382	44097048	G	A	0.8462	0.621	7.74E-04
20	BICF2P1394766	44400207	A	G	0.8231	0.5806	8.01E-04
20	BICF2P148086	44473374	G	C	0.8231	0.5806	8.01E-04
20	BICF2P180129	44738837	T	C	0.8231	0.5806	8.01E-04
20	BICF2P712446	44671609	T	A	0.8231	0.5806	8.01E-04
1	BICF2S2349549	111809273	T	C	0.8615	0.6774	8.12E-04
20	BICF2P305117	33486951	C	A	0.7692	0.5164	8.39E-04
20	BICF2P410834	46575321	A	G	0.8923	0.7581	8.49E-04
20	BICF2P515331	46586368	C	T	0.8923	0.7581	8.49E-04
20	BICF2P543637	46570835	C	T	0.8923	0.7581	8.49E-04
20	BICF2S23056251	46343056	G	A	0.8923	0.7581	8.49E-04
20	BICF2S2397814	46596719	G	A	0.8923	0.7581	8.49E-04
20	BICF2S2411367	46367754	C	T	0.8923	0.7581	8.49E-04
2	BICF2P20779	38693470	G	A	0.8231	0.629	8.54E-04
2	BICF2P218913	38653901	A	C	0.8231	0.629	8.54E-04
2	BICF2P52967	38710317	T	C	0.8231	0.629	8.54E-04
2	BICF2S23340033	38665751	C	T	0.8231	0.629	8.54E-04
10	BICF2P722107	25703920	T	C	0.4769	0.2661	8.57E-04
29	BICF2P275153	7066011	T	C	0.93077	0.8065	8.88E-04
36	BICF2P888360	29865343	C	T	0.8154	0.5887	9.22E-04
2	BICF2P1177232	41929907	A	G	0.8462	0.6371	9.23E-04
2	BICF2P601492	41925417	T	C	0.8462	0.6371	9.23E-04
20	BICF2P1081825	39156399	C	G	0.8231	0.5806	9.31E-04
20	BICF2S23749844	39354310	G	A	0.8231	0.5806	9.31E-04
20	TIGRP2P274409	39317496	C	A	0.8231	0.5806	9.31E-04
2	BICF2P832726	41781459	G	A	0.8154	0.5968	9.43E-04
2	BICF2P1164239	29109220	G	A	0.7692	0.5726	9.43E-04
2	BICF2G630673509	17809319	T	G	0.8615	0.96774	9.56E-04
6	BICF2S2373412	58405661	G	C	0.7385	0.5081	9.69E-04
20	BICF2S23344904	39351635	C	T	0.8231	0.575	9.75E-04

33	TIGRP2P385894	12755495	T	C	0.93846	0.7581	9.79E-04
20	BICF2P1100219	45555190	A	G	0.8231	0.5565	9.94E-04
20	BICF2P186258	45693884	T	C	0.8231	0.5565	9.94E-04
20	BICF2P193872	45704079	T	C	0.8231	0.5565	9.94E-04
20	BICF2P474075	45547322	A	G	0.8231	0.5565	9.94E-04
20	BICF2P728963	45622457	A	C	0.8231	0.5565	9.94E-04
20	BICF2P921104	45535898	T	C	0.8231	0.5565	9.94E-04
20	BICF2S23413507	45638263	G	A	0.8231	0.5565	9.94E-04
20	BICF2P247805	38507160	C	T	0.8231	0.6048	9.95E-04
29	BICF2P616092	7197512	T	G	0.93077	0.7984	1.01E-03
20	BICF2P1056026	49003875	A	G	0.7308	0.4839	1.01E-03
20	BICF2P1268674	48945007	G	A	0.7308	0.4839	1.01E-03
20	BICF2P1389617	48930946	T	C	0.7308	0.4839	1.01E-03
2	BICF2P1109306	35912638	A	G	0.7538	0.5565	1.02E-03
2	BICF2P398615	35899773	C	T	0.7538	0.5565	1.02E-03
20	BICF2S23449078	46959950	T	G	0.6385	0.3952	1.05E-03
20	BICF2P384455	48206831	G	A	0.8846	0.7258	1.06E-03
34	BICF2G630461632	22554159	T	G	0.4692	0.2823	1.06E-03
34	BICF2G630461634	22545730	C	T	0.4692	0.2823	1.06E-03
34	BICF2G630461644	22535208	A	G	0.4692	0.2823	1.06E-03
34	BICF2P595821	22301058	A	G	0.4692	0.2258	1.06E-03
20	BICF2P1294383	38524299	A	G	0.8231	0.6129	1.07E-03
34	BICF2P228264	21795887	G	A	0.5	0.2903	1.09E-03
6	G421F63S108	64950274	A	G	0.6462	0.4032	1.12E-03
6	BICF2P124875	58363908	A	G	0.7385	0.5082	1.12E-03
20	BICF2P249939	37884109	T	C	0.98462	0.871	1.13E-03
32	BICF2S23438286	27039372	T	C	0.8462	0.96774	1.16E-03
2	BICF2P351346	27632525	T	C	0.7615	0.5403	1.17E-03
2	BICF2P820410	27603571	T	C	0.7615	0.5403	1.17E-03
17	BICF2S23550856	58489749	C	T	0.8385	0.6855	1.17E-03
20	BICF2S236547	46720507	T	C	0.8846	0.75	1.18E-03
20	G1113F42S23	46820316	G	A	0.8846	0.75	1.18E-03
2	BICF2P1112438	36296335	C	T	0.7308	0.5242	1.20E-03
29	TIGRP2P363515	4679068	A	G	0.8308	0.6532	1.23E-03
6	BICF2P1134114	65119926	G	T	0.7231	0.5081	1.24E-03

S1b table)
