# Supplementary material for: Genome-Wide Association Study of Golden Retrievers Identifies Germ-Line Risk Factors Predisposing to Mast Cell Tumours
Source: PLoS Genet. 2015 Nov 20;11(11):e1005647. doi: 10.1371/journal.pgen.1005647 (PMC4654484; doi:10.1371/journal.pgen.1005647)
Supplement: S2 Table — Allele frequencies for cases and controls. p-values and permutated p-values (1.000.000 permutations) are shown. P-values for SNPs, which were included in the GWAS and repeated in the fine mapping are shown. Only the 20 most associated iplex SNPs are shown. (PDF) [file pgen.1005647.s011.pdf]

SNP	Risk allele	Freq case/control	p-value	Permuted p-value 1,000,000	GWAS p-value
chr14:14644897	C	0.798 / 0.562	6.36E-08	9.0000E-6	7.665e-07
chr14:14666424	C	0.724 / 0.483	1.59E-07	0.0001	
chr14:14685771	G	0.796 / 0.566	1.62E-07	0.0001	
chr14:14685543	C	0.803 / 0.576	1.67E-07	0.0002	7.665e-07
chr14:14685602	G	0.825 / 0.608	4.28E-07	0.0003	
chr14:14682089	T	0.781 / 0.557	4.96E-07	0.0003	
chr14:14714009	G	0.784 / 0.579	4.08E-06	0.0006	3.159E--7
chr14:14923231	A	0.551 / 0.344	5.33E-06	0.0007	
chr14:14840602	C	0.586 / 0.381	7.83E-06	0.0008	
chr14:14869184	A	0.556 / 0.352	8.17E-06	0.0009	
chr14:14767603	C	0.586. 0.383	1.00E-05	0.0011	
chr14:14840707	C	0.589 / 0.385	1.21E-05	0.0014	
chr14:14767966	C	0.582 / 0.387	2.32E-05	0.0041	
chr14:14653880	C	0.601 / 0.406	2.34E-05	0.0041	
chr14:14664532	T	0.581 / 0.395	4.92E-05	0.0066	
chr14:14661891	G	0.571 / 0.388	6.55E-05	0.0075	
chr14:14866084	G	0.667 / 0.483	7.02E-05	0.0077	
chr14:14827179	C	0.667 / 0.496	3.00E-04	0.0161	
chr20:42080147	T	0.065 / 0.010	7.00E-04	0.0441	
chr14:14610095	T	0.183 / 0.118	0.0512	0.6967	

S2 table)
