# Supplementary material for: Genome-Wide Association Study of Golden Retrievers Identifies Germ-Line Risk Factors Predisposing to Mast Cell Tumours
Source: PLoS Genet. 2015 Nov 20;11(11):e1005647. doi: 10.1371/journal.pgen.1005647 (PMC4654484; doi:10.1371/journal.pgen.1005647)
Supplement: S3 Table — Allele frequencies. p-values and permutated p-values (1.000.000 permutations) are showing for the European (EU) analysis and the combined European and United States analysis. GWAS p-values for SNPs, which were included in the GWAS and repeated in the iplex are shown. (PDF) [file pgen.1005647.s012.pdf]

SNP	Assoc Allele	Case/Control Frequencies	p-value iplex EU	Permuted p-value 1.000.000 EU	GWAS EU	p-value iplex EU/US	Permuted p-value 1000000 EU/US
chr14:14610095	T	0.135/ 0.103	7.48E-01	1.00E+00		1.87E-01	9.79E-01
chr14:14644897	C	0.617/ 0.486	5.66E-01	1.00E+00	2.38E-01	4.00E-04	3.70E-02
chr14:14653880	C	0.408/ 0.294	2.92E-02	6.07E-01		1.20E-03	8.08E-02
chr14:14661891	G	0.375/ 0.281	9.92E-02	9.66E-01		6.30E-03	2.38E-01
chr14:14664532	T	0.381/ 0.286	9.92E-02	9.66E-01		5.80E-03	2.21E-01
chr14:14666424	C	0.466/ 0.349	1.29E-01	9.85E-01		1.30E-03	8.92E-02
chr14:14682089	T	0.615/ 0.477	3.12E-01	1.00E+00		3.00E-04	3.04E-02
chr14:14685543	C	0.619/ 0.498	6.56E-01	1.00E+00	2.32E-01	1.00E-03	6.97E-02
chr14:14685602	G	0.661/ 0.525	1.97E-01	1.00E+00		2.00E-04	2.70E-02
chr14:14685771	G	0.617/ 0.490	5.37E-01	1.00E+00		6.00E-04	4.98E-02
chr14:14714009	G	0.622/ 0.503	6.10E-01	1.00E+00	2.66E-01	1.90E-03	1.13E-01
chr14:14767603	C	0.383/ 0.279	1.63E-01	9.98E-01		2.70E-03	1.35E-01
chr14:14767966	C	0.379/ 0.280	1.63E-01	9.98E-01		4.50E-03	1.90E-01
chr14:14827179	C	0.539/ 0.432	3.40E-01	1.00E+00		4.90E-03	1.98E-01
chr14:14840602	C	0.387/ 0.283	3.28E-01	1.00E+00		2.70E-03	1.36E-01
chr14:14840707	C	0.387/ 0.286	3.28E-01	1.00E+00		4.20E-03	1.81E-01
chr14:14866084	G	0.541/ 0.430	4.25E-01	1.00E+00		2.90E-03	1.40E-01
chr14:14869184	A	0.365/ 0.256	1.55E-01	9.96E-01		1.40E-03	9.11E-02
chr14:14923231	A	0.366/ 0.250	6.26E-02	8.96E-01		6.00E-04	5.08E-02
chr20:41512961	C	0.538/ 0.401	8.94E-05	9.40E-03		3.00E-04	2.99E-02
chr20:41543010	A	0.609/ 0.498	7.01E-05	8.70E-03		2.70E-03	1.36E-01
chr20:41614101	A	0.604/ 0.502	3.00E-04	1.90E-02		5.70E-03	2.18E-01
chr20:41614453	G	0.877/ 0.857	9.21E-01	1.00E+00		4.47E-01	1.00E+00
chr20:41662902	A	0.599/ 0.502	4.00E-04	2.94E-02		9.00E-03	2.92E-01
chr20:41712898	A	0.644/ 0.514	9.15E-05	1.91E-02		4.00E-04	3.89E-02
chr20:41732334	T	0.645/ 0.510	2.39E-05	3.50E-03		3.00E-04	3.03E-02
chr20:41733976	G	0.642/ 0.517	7.11E-05	8.70E-03		7.00E-04	5.36E-02
chr20:41828740	T	0.519/ 0.365	2.18E-05	3.40E-03		3.37E-05	8.10E-03
chr20:41909338	C	0.663/ 0.553	4.00E-04	2.99E-02		2.60E-03	1.35E-01
chr20:41927603	T	0.590/ 0.431	5.05E-06	1.60E-03		2.00E-04	2.53E-02
chr20:41930509	G	0.594/ 0.443	7.36E-05	8.90E-03	2.13E-06	3.93E-05	8.80E-03
chr20:41933198	G	0.589/ 0.442	1.00E-04	1.24E-02		7.32E-05	1.22E-02
chr20:41951828	T	0.590/ 0.445	1.00E-04	1.24E-02	2.13E-06	9.28E-05	1.46E-02
chr20:41970787	G	0.667/ 0.553	3.00E-04	2.54E-02		1.80E-03	1.06E-01
chr20:41972158	C	0.719/ 0.595	1.20E-03	5.86E-02		4.00E-04	4.15E-02
chr20:41972956	C	0.595/ 0.440	7.36E-05	8.90E-03		2.81E-05	6.90E-03
chr20:41987996	G	0.593/ 0.442	9.31E-05	9.70E-03		4.90E-05	9.90E-03

chr20:41990290	C	0.593/ 0.446	9.31E-05	9.70E-03		8.19E-05	1.30E-02
chr20:41993220	T	0.589/ 0.442	3.00E-04	2.52E-02		7.22E-05	1.21E-02
chr20:41996894	C	0.600/ 0.450	2.86E-05	4.60E-03		6.33E-05	1.11E-02
chr20:42060186	T	0.541/ 0.377	1.41E-05	2.80E-03		1.06E-05	3.10E-03
chr20:42080147	T	0.367/ 0.114	2.03E-15	0.00E+00		2.18E-16	0.00E+00
chr20:42108401	A	0.668/ 0.536	8.79E-05	9.40E-03		4.00E-04	3.65E-02
chr20:42111613	G	0.641/ 0.530	1.00E-03	5.46E-02		3.20E-03	1.49E-01
chr20:42114307	A	0.669/ 0.539	3.00E-04	1.92E-02		3.00E-04	3.60E-02
chr20:42115073	G	0.661/ 0.532	8.79E-05	9.40E-03		4.00E-04	4.03E-02
chr20:42117345	T	0.666/ 0.536	7.01E-05	8.70E-03		4.00E-04	4.05E-02
chr20:42131456	A	0.577/ 0.400	7.71E-06	2.30E-03		1.65E-06	1.00E-03
chr20:42131853	G	0.646/ 0.507	1.34E-05	2.60E-03		2.00E-04	2.52E-02
chr20:47886402	C	0.347/ 0.240	3.00E-04	1.97E-02		1.60E-03	9.75E-02
chr20:47899650	A	0.351/ 0.235	2.00E-04	1.55E-02		6.00E-04	4.99E-02
chr20:48051957	G	0.437/ 0.349	6.16E-02	8.84E-01		1.52E-02	3.82E-01
chr20:48052681	C	0.352/ 0.234	6.82E-05	8.50E-03		5.00E-04	4.20E-02
chr20:48055355	G	0.418/ 0.338	6.56E-02	9.03E-01		2.55E-02	5.21E-01
chr20:48056097	G	0.152/ 0.083	5.07E-05	7.50E-03		3.60E-03	1.63E-01
chr20:48056581	T	0.429/ 0.346	8.60E-02	9.46E-01		2.05E-02	4.63E-01
chr20:48059078	T	0.355/ 0.231	7.16E-05	8.80E-03		2.00E-04	2.56E-02
chr20:48060281	G	0.437/ 0.347	8.31E-02	9.44E-01		1.38E-02	3.62E-01
chr20:48062375	C	0.433/ 0.344	6.41E-02	8.99E-01		1.43E-02	3.67E-01
chr20:48062389	G	0.420/ 0.347	1.56E-01	9.96E-01		4.50E-02	6.59E-01
chr20:48062854	G	0.357/ 0.236	2.00E-04	1.55E-02		3.00E-04	3.25E-02
chr20:48072724	A	0.382/ 0.281	1.80E-03	8.47E-02		3.80E-03	1.68E-01
chr20:48111692	T	0.349/ 0.228	6.82E-05	8.50E-03		3.00E-04	3.33E-02
chr20:48112205	T	0.351/ 0.232	1.00E-04	1.21E-02		4.00E-04	3.81E-02
chr20:48117256	A	0.343/ 0.231	2.00E-04	1.42E-02		8.00E-04	5.99E-02
chr20:48130277	G	0.418/ 0.338	1.13E-01	9.79E-01		2.55E-02	5.21E-01
chr20:48150406	G	0.386/ 0.297	1.34E-02	3.93E-01		1.07E-02	3.18E-01
chr20:48158297	C	0.387/ 0.291	1.18E-02	3.76E-01		6.00E-03	2.27E-01
chr20:48159029	A	0.396/ 0.290	5.20E-03	1.97E-01		2.30E-03	1.26E-01
chr20:48160311	C	0.414/ 0.334	6.95E-02	9.18E-01		2.51E-02	5.17E-01
chr20:48162500	G	0.388/ 0.290	6.90E-03	2.41E-01		5.00E-03	2.00E-01
chr20:48259767	T	0.415/ 0.298	1.20E-03	6.11E-02		8.00E-04	6.15E-02
chr20:48260231	G	0.422/ 0.304	1.90E-03	8.73E-02		9.00E-04	6.79E-02
chr20:48377580	A	0.353/ 0.237	2.00E-04	1.51E-02		5.00E-04	4.64E-02
chr20:48429591	A	0.388/ 0.301	6.90E-03	2.42E-01		1.33E-02	3.55E-01
chr20:48437593	T	0.418/ 0.340	8.29E-02	9.44E-01		3.65E-02	6.05E-01
chr20:48520099	T	0.347/ 0.236	1.00E-04	1.17E-02		9.00E-04	6.64E-02
chr20:48599799	A	0.357/ 0.240	6.48E-05	8.40E-03	4.33E-07	5.00E-04	4.65E-02
chr20:48601051	C	0.500/ 0.416	1.43E-01	9.90E-01		2.28E-02	4.93E-01

chr20:48650307	A	0.372/ 0.306	3.39E-02	6.71E-01		7.97E-02	8.29E-01
chr20:48704449	C	0.445/ 0.364	6.96E-02	9.18E-01		2.49E-02	5.15E-01
chr20:48743303	G	0.320/ 0.269	8.13E-01	1.00E+00		1.27E-01	9.22E-01
chr20:48743330	T	0.451/ 0.367	7.61E-02	9.29E-01		2.18E-02	4.75E-01
chr20:48744441	G	0.459/ 0.371	7.31E-02	9.21E-01		1.73E-02	4.14E-01
chr20:48756142	G	0.431/ 0.324	6.10E-03	2.19E-01		3.20E-03	1.49E-01
chr20:48756169	C	0.420/ 0.322	1.67E-02	4.61E-01		6.20E-03	2.33E-01
chr20:48802224	A	0.435/ 0.363	1.54E-01	9.95E-01		4.78E-02	6.76E-01
chr20:48804130	G	0.451/ 0.364	9.12E-02	9.55E-01		1.67E-02	4.04E-01
chr20:48811857	A	0.451/ 0.360	5.57E-02	9.55E-01		1.25E-02	3.47E-01
chr20:48841374	G	0.395/ 0.292	1.09E-02	3.65E-01		3.40E-03	1.53E-01
chr20:48855117	A	0.982/ 0.957	3.11E-02	6.38E-01		5.74E-02	7.34E-01
chr20:48906397	T	0.406/ 0.298	1.36E-02	3.94E-01		2.30E-03	1.27E-01
chr20:49051904	C	0.369/ 0.269	1.90E-03	8.67E-02		3.50E-03	1.57E-01
chr20:49201505	G	0.355/ 0.223	2.08E-05	3.40E-03	8.06E-07	8.05E-05	1.29E-02
chr20:49479706	A	0.892/ 0.876	7.61E-02	9.30E-01		5.09E-01	1.00E+00
chr20:49671452	G	0.466/ 0.382	4.35E-02	7.65E-01		2.18E-02	4.76E-01
chr20:49687024	G	0.357/ 0.231	3.00E-04	1.95E-02		2.00E-04	2.18E-02
chr20:49691940	A	0.344/ 0.221	3.00E-04	1.91E-02		2.00E-04	2.67E-02

S3 table)
